# Indirect Fitness Benefits Enable the Spread of Host Genes Promoting Costly Transfer of Beneficial Plasmids

**DOI:** 10.1371/journal.pbio.1002478

**Published:** 2016-06-07

**Authors:** Tatiana Dimitriu, Dusan Misevic, Chantal Lotton, Sam P. Brown, Ariel B. Lindner, François Taddei

**Affiliations:** 1 Institut National de la Santé et de la Recherche Médicale, U1001, Université Paris Descartes, Sorbonne Paris Cité, Paris, France; 2 Georgia Institute of Technology, School of Biology, Atlanta, Georgia, United States of America; University of Lausanne, SWITZERLAND

## Abstract

Bacterial genes that confer crucial phenotypes, such as antibiotic resistance, can spread horizontally by residing on mobile genetic elements (MGEs). Although many mobile genes provide strong benefits to their hosts, the fitness consequences of the process of transfer itself are less clear. In previous studies, transfer has been interpreted as a parasitic trait of the MGEs because of its costs to the host but also as a trait benefiting host populations through the sharing of a common gene pool. Here, we show that costly donation is an altruistic act when it spreads beneficial MGEs favoured when it increases the inclusive fitness of donor ability alleles. We show mathematically that donor ability can be selected when relatedness at the locus modulating transfer is sufficiently high between donor and recipients, ensuring high frequency of transfer between cells sharing donor alleles. We further experimentally demonstrate that either population structure or discrimination in transfer can increase relatedness to a level selecting for chromosomal transfer alleles. Both mechanisms are likely to occur in natural environments. The simple process of strong dilution can create sufficient population structure to select for donor ability. Another mechanism observed in natural isolates, discrimination in transfer, can emerge through coselection of transfer and discrimination alleles. Our work shows that horizontal gene transfer in bacteria can be promoted by bacterial hosts themselves and not only by MGEs. In the longer term, the success of cells bearing beneficial MGEs combined with biased transfer leads to an association between high donor ability, discrimination, and mobile beneficial genes. However, in conditions that do not select for altruism, host bacteria promoting transfer are outcompeted by hosts with lower transfer rate, an aspect that could be relevant in the fight against the spread of antibiotic resistance.

## Abstract 2

Altruistic host bacteria can preferentially enhance the horizontal transfer of beneficial plasmids (such as those conferring antibiotic resistance or virulence) to others of their kind.

## Author Summary

In bacteria, genes can move between cells, sometimes with the donor host cell actively involved in the gene transfer mechanisms. This movement of genes is called horizontal gene transfer, and it increases the prevalence of mobile genes in bacterial populations. However, it is not clear if donor host cells benefit from gene spread, or are simply exploited by selfish genes. Here, we show with both modelling and experiments that for the donor host, investing in the transfer of beneficial genes—such as those conferring antibiotic resistance—can be understood as an altruistic behaviour. This behaviour is costly to the donor but beneficial to recipients and can be selected for if a sufficient proportion of recipient cells share the donors’ transfer allele. Preferential transfer from donors towards recipients that share this allele occurs when dispersal is limited or if discrimination mechanisms are present. Our work suggests that both processes are likely to be widespread in nature, promoting horizontal gene spread by host donor cells. As many antimicrobial resistance and virulence genes are mobile, our work further implies that the spread of harmful traits among human pathogens may be modulated by host bacteria in a direction that depends on the bacterial ability to transfer the traits specifically to their kind.

## Introduction

MGEs such as plasmids or phages are defined by their ability to undergo horizontal gene transfer (HGT) between bacterial hosts [1], and are widespread in nature. Genes present on MGEs often affect their hosts’ fitness in a specific environment [2]. Particularly, many mobile genes increase virulence or antibiotic resistance and thus have harmful consequences on human health. Antibiotic resistance genes are enriched on plasmids [3], leading to their fast spread among bacterial species via horizontal transfer [4]. Genes coding for secreted proteins, often involved in virulence, are also enriched on MGEs promoting cooperative secretion [5,6]. In order to better combat the medical issues arising from horizontal transfer, we must understand the selective pressures acting on gene mobility. The population dynamics and evolution of transfer have mostly been studied by focusing on MGEs themselves [7]; however, transfer is influenced not only by MGE genes, but also by genes of the bacterial host chromosome. Both donor [8] and recipient cells [9,10] can regulate transfer, with different donor and recipient genetic backgrounds resulting in as much as eight orders of magnitude variance in the transfer rates for the same plasmid [11,12]. Thus, to fully understand the evolution of horizontal gene spread and the natural variation in transfer rates among hosts, we must consider the selective pressures acting on hosts.

On one side, horizontal transfer confers varied and often extreme costs onto the bacterial host. Phage mobility usually requires host cell lysis that leads to death, while plasmid transfer through conjugation renders host cells sensitive to male-specific phages [13] and decreases the host's growth rate and fitness [14,15]. Because of these costs, horizontal transfer has classically been considered as a selfish trait of parasitic MGEs, selected as it favours their spread [7]. Direct support for transfer being a purely costly trait to the host came from studies of plasmid–host coevolution, where host genes that decrease transfer were selected [16]. On the other side, it has also been suggested that HGT could benefit the host because of the transfer of accessory genes not directly involved in MGE maintenance and transfer. Indeed, MGEs are often thought to constitute a communal pool of genes [2], a flexible genome [17] that can be quickly shuffled by HGT in response to environmental changes, making host populations more robust [18]. In this view, HGT is beneficial to the host population because it allows cells to share beneficial traits and provides diversity at the population level. However, it is not clear that these proposed benefits are sufficient for HGT to be favoured by the host. Traits advantageous at the group level—here the maintenance of a communal pool of genes—are not necessarily selected for at the individual level, especially when individuals can benefit from others that invest in the trait while not paying the cost of investing themselves [19]. Indirect, population-wide benefits alone are not necessarily sufficient to explain the selection of host genes promoting costly transfer [20]. The ability to receive genes can clearly be directly selected for when these genes enhance individual fitness: for instance, CRISPR immunity against antibiotic resistance plasmids, a form of HGT resistance, was rapidly lost in the presence of antibiotics when receiving plasmids was beneficial to the host [21]. On the contrary, the ability to donate genes need not be selected, as the donor cell does not directly benefit from transferring genes to neighbouring recipients.

To quantitatively understand HGT, the selection acting on donor ability must be analysed in a social context, taking into account both the costs and benefits transfer bestows onto donor and recipient hosts. Here, we theoretically and experimentally analyse the evolution of host genes controlling plasmid transfer. We show that from the host side, transfer represents a form of altruism: actors pay a cost of investing in transmission and deliver a benefit to recipients of beneficial mobile elements. Altruistic donation of MGEs can be maintained when transfer is sufficiently biased towards cells sharing donation alleles, increasing the donor allele inclusive fitness. This bias can arise in structured populations or by an association between transfer and discrimination alleles. Fitness gains due to the transfer of mutualistic plasmids further select for genotypes where donor ability alleles, discrimination alleles, and mutualistic plasmids are associated.

## Results

### Schematic Model

We first perform a qualitative analysis to identify if and in which conditions a strain with high donor ability can be selected. We model the fitness of nonmobile host genes controlling donor ability for a given plasmid using a neighbour-modulated fitness approach that partitions fitness into the effects of an individual’s own genotype and those of social neighbours [22,23]. We consider a population of bacteria structured in an infinite number of patches [24] and model a simplified life cycle with nonoverlapping patch generations, in which the following processes occur successively [25,26]: founding, reproduction, transfer, selection, and dispersal (see S1 Text for details).

A cell *i* in patch *j* is characterized by three traits: plasmid carriage *p*_*ij*_ (*p*_*ij*_ = 1 for plasmid-bearing cells and 0 for plasmid-free cells), donor ability *q*_*ij*_ and recipient ability *s*_*ij*_. Successful transfer is controlled by three factors: the probability of contact between plasmid-bearing and plasmid-free cells, the donor ability of plasmid-bearing cells, and the recipient ability of plasmid-free cells. We assume that plasmid and host traits are distributed independently in the starting population so that the cell's donor ability *q*_*ij*_ is independent from its initial plasmid content *p*_*ij*_. Initially uninfected cells become infected with a probability proportional to the patch level frequency of plasmid-bearing cells modulated by their own recipient ability and by the average patch donor ability. A cell *i* in patch *j* will thus be modified by transfer with the probability (1 − *p*_*ij*_) *p*_*j*_ *q*_*j*_*s*_*ij*_.

Plasmid presence has an effect *e*_*p*_ on the host cell, and we can express the plasmid effect on host fitness as *e*_*p*_ *p*_*ij*_. The cost of donor ability is *c*_*q*_ leading to an effect of transfer on host fitness that is proportional to donor ability, experienced only by cells bearing plasmids before transfer, and equal to −*p*_*ij*_ *c*_*q*_ *q*_*ij*_. Donor ability is costly independently of actual transfer efficiency, modelling the effect of expressing the transfer machinery (which happens even in the absence of successful transfer).

The fitness of an individual founding cell *i* in patch *j*, measured over the patch life cycle, is noted by *W*_*ij*_. With *W*_0_ being the basal host fitness, we obtain (see S1 Text):

$$W_{ij}=W_{0}+e_{p}\;[p_{ij}+(1-p_{ij})p_{j}\;q_{j}s_{ij}]-p_{ij}\;c_{q}q_{ij}$$ (1)

To understand selection acting on donor ability *q*, we apply the Price equation [27,28] to Eq (1). We obtain the regression coefficient between fitness and donor ability, *β*(*W*_*ij*_,*q*_*ij*_), that describes the effect of donor ability on fitness (Eq 2). We provide the derivation of Eq (2) and a detailed analysis in S1 Text.

$$\beta (W_{ij},q_{ij})=e_{p}\;E_{j}[p_{j}(1-p_{j})]\beta (q_{j}s_{ij},q_{ij})-p\;c_{q}$$ (2)

The *E*_*j*_[*p*_*j*_(1 − *p*_*j*_)] term describes the effect of patch composition on the efficiency of plasmid transfer: transfer events are more likely when both plasmid-bearing and plasmid-free cells are abundant within each patch. *β*(*q*_*j*_*s*_*ij*_,*q*_*ij*_) is a regression coefficient between individual donor ability *q*_*ij*_ and the product of individual recipient ability with patch-level donor ability. It corresponds to the relatedness between plasmid donor and recipient cells, noted by *R*_*q*_, at the locus determining donor ability (see S1 Text for a detailed analysis): *R*_*q*_ is higher when donor cells preferentially encounter recipients that share their donation allele and when transfer is more successful towards those cells. *R*_*q*_ thus determines how much a donor cell transfers plasmids to individuals bearing the same donation allele because of population structure and specificity in transfer. Finally, *p c*_*q*_ is the average cost of transfer for the donor genotype: high donor ability is costly to the proportion of cells that bear plasmids and express their transfer machinery.

An increase in donor ability is selected for when it is correlated with increase in fitness, namely when *β*(*W*_*ij*_,*q*_*ij*_) > 0, which combined with Eq (2) leads to the following condition:

$$e_{p}\;E_{j}[p_{j}(1-p_{j})]R_{q}>pc_{q}$$ (3)

Eq 3 is a form of Hamilton’s rule [29], which postulates that a cooperative allele is selected for when its indirect benefits, weighted by relatedness among actors and recipients, outweigh its direct cost, maximizing its inclusive fitness (fitness inclusive of alleles present in other individuals). Applied here to donor ability, the indirect benefits are the benefits of plasmids to the recipient cells after transfer *e*_*p*_ *E*_*j*_[*p*_*j*_(1 − *p*_*j*_)], and the direct cost is the cost of donor ability for cells bearing plasmids *p c*_*q*_. *R*_*q*_ is the relatedness at the donor ability locus among donor and recipient cells of plasmid transfer. High, positive *R*_*q*_ implies that most of transfer events from cells with high donor ability will be directed towards recipients sharing their donation allele. On the contrary, negative *R*_*q*_ means that transfer will be biased towards cells with a different allele than the one carried by the donor. Thus, a high donor ability allele can be selected even when individually costly, when transfer maximizes its inclusive fitness through plasmid effects on recipient cells. We note that relatedness in bacteria can vary across loci [30], as it can be modified in a locus-specific way by mutation [31] or HGT [5,6]. Thus, unlike relatedness arising from genealogical kinship in sexually reproducing organisms, it will not necessarily tend to be the same across the genome. To underline this and avoid any potential semantic confusion, we follow nomenclature defined already in [30] and consider that cells that specifically share alleles at the locus of interest (plasmid donation) are cells of the same kind but not necessarily kin.

*R*_*q*_ will be positive when donors preferentially transfer plasmids to recipients of their kind. Positive relatedness generally arises through the combination of two processes: limited dispersal and discrimination mechanisms [29,32]. Here, limited dispersal is due to patch structure: the correlation between *q*_*j*_ and *q*_*ij*_ is governed by the initial repartition of genotypes among patches, with no migration before transfer occurs. Positive relatedness can arise from strong population bottlenecks leading to stochastic variations in founding cell frequencies among patches, followed by clonal reproduction [33]. Effective discrimination in transfer also leading to positive relatedness arises if *s*_*ij*_ and *q*_*ij*_ are positively correlated, with genotypes with high donor ability having higher recipient ability than average, or if donors have a way to direct transfer specifically to their kind (see S1 Text for discussion). Alternatively, negative relatedness can arise if *s*_*ij*_ and *q*_*ij*_ are negatively correlated, leading to preferential transfer to cells bearing a different donation allele.

We can distinguish two scenarios for the effect and selection of transfer depending on the plasmid effects on the host cell. In the first case, the transferred plasmid is mutualistic with its host (*e*_*p*_ > 0), for instance conferring antibiotic resistance: transfer is therefore an altruistic behaviour [29] with a direct cost of performing transfer and indirect benefits through the plasmid benefits in recipient cells. Transfer is selected if *R*_*q*_ is positive and sufficiently high: *R*_*q*_ > *p c*_*q*_ / (*e*_*p*_ *E*_*j*_[*p*_*j*_(1 − *p*_*j*_)]). In the second case, the transferred plasmid is parasitic (*e*_*p*_ < 0): donor ability for parasitic plasmids, decreasing the fitness of recipient cells, is selected if *R*_*q*_ is negative and sufficiently low: *R*_*q*_ < *p c*_*q*_ / (*e*_*p*_ *E*_*j*_[*p*_*j*_(1 − *p*_*j*_)]). This would be a case of spiteful behaviour [32,34]. Specific population structure or discrimination processes are required to produce negative relatedness, and spite is thus thought to be less common than altruism [34].

**Fig 1:**
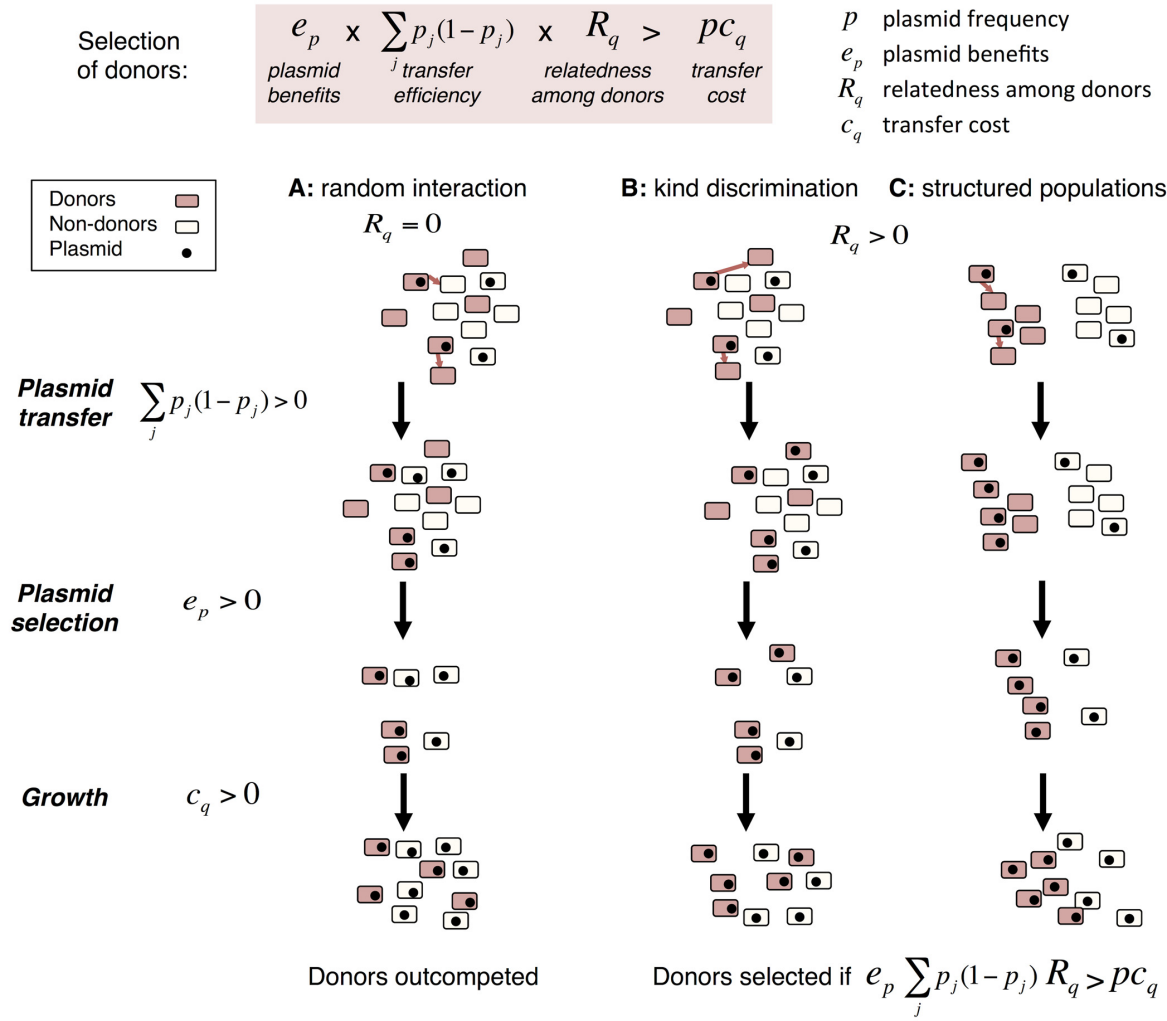
Graphical representation of different scenarios for the selection of transfer as an altruistic trait. In this simplified diagram, we follow a strain with high donor ability (red) in competition with another strain with no donor ability (white). Some cells of both strains bear an antibiotic resistance plasmid (black dots) that donors can transfer (red arrows) to a cell of either type, as long as it is plasmid-free. Our model predicts that donors are selected for when the red-framed equation is true (Eq 3, see main text). For clarity, we assume three sequential steps: (1) transfer, whose recipients depend on relatedness at the donor ability locus (*R*_*q*_) and whose efficiency depends on plasmid frequency within patches *p*_*j*_; (2) antibiotic selection, where only plasmid-bearing cells survive (*e*_*p*_ > 0); and finally, (3) cell growth after selection, where donor cells experience a cost *c*_*q*_ and grow more slowly. We describe three possible scenarios, depending on the properties of transfer and its effects on relatedness at the donation locus. **A:** In the absence of discrimination in transfer or population structure, relatedness among donors and recipients is null, and transfer occurs with the same efficiency towards all cells. **B:** In the presence of discrimination in transfer, good donors transfer plasmids specifically to their kind. **C:** In structured populations, good donors are surrounded by their kind, to which they preferentially transfer plasmids even in the absence of discrimination. In all scenarios, donor cells experience the cost of expressing the transfer machinery during growth. However, only in **B** and **C** does transfer bias lead to an enrichment of plasmids in the donor strain after transfer, which can compensate for donor ability cost when plasmids are selected for.

We focus here on the transfer of mutualistic MGEs and more specifically on antibiotic resistance plasmids that allow their hosts to grow when antibiotics are present. The main prediction arising from our model is that donor ability for these mutualistic plasmids is an altruistic trait, counterselected if transfer occurs indiscriminately towards any cell, but selected for when plasmid donors and recipients share donation alleles. We present the model graphically in Fig 1, focusing on the three relevant scenarios affecting relatedness: random interactions between individuals (Fig 1A), discrimination in transfer (Fig 1B), and structured populations (Fig 1C). We next test the model’s predictions with both simulations and experiments, performing competition assays between strains with varying donor ability in order to investigate quantitatively if and how much selection favours donor ability in biologically realistic settings.

### Selection for Donor Ability through Discrimination in Transfer

**Fig 2:**
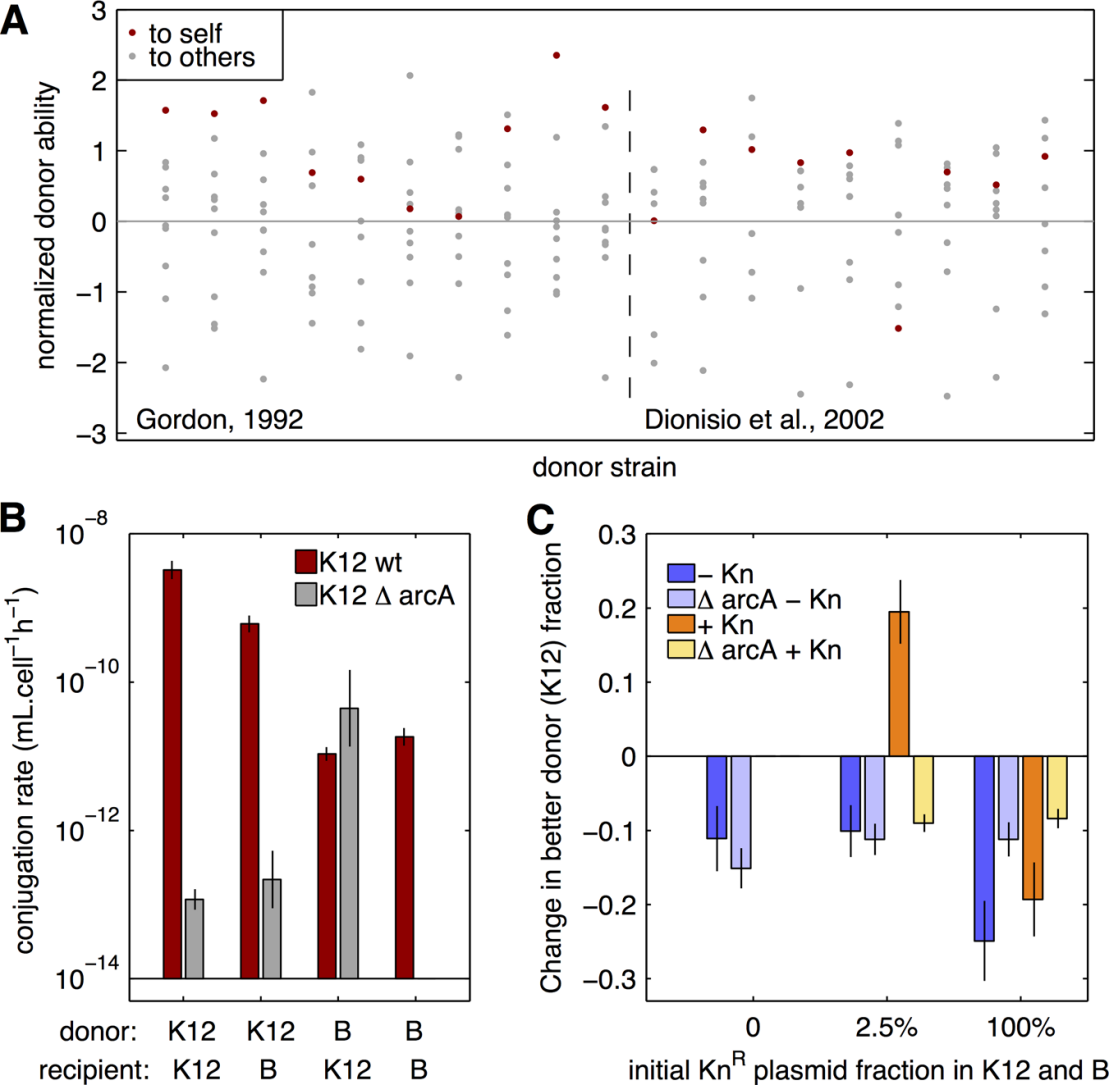
Selection for plasmid transfer through discrimination of recipients. **A: Donor ability to self and others among natural isolates.** A normalized donor ability for the R1 plasmid for couples of donor and recipient strains was obtained by correcting conjugation rates from [11] (left of the dashed line) and [12] (right of the dashed line) datasets by the average strain donor ability across all recipient strains tested. Points in red indicate donor ability measured from a strain to itself. **B: Conjugation rates between *E*. *coli* K12 and B strains.** Conjugation rates were measured in the same growth conditions as for Fig 2C competitions. Donors and recipients were mixed during exponential growth (optical density [OD] = 0.2), then donor (*D*), recipient (*R*), and transconjugant (*T*) densities were measured by plating. Conjugation rates were computed as $\gamma =\frac{T}{DRt}$ (mL.cell^-1^.h^_1^), and are shown as geometric means ± standard error of the mean (SEM) (*N* ≥10). Red bars show conjugation data obtained with the wild type K12 strain, grey bars show conjugation using the K12ΔarcA mutant. **C: Competition between K12 and B strains with antibiotic resistance transfer.** The change in frequency of K12 strain is shown in competition with B strain in a single well-mixed population, in the absence (blue) or presence (yellow) of antibiotic selection at the end of competition, and for different initial proportions of the R1-19 plasmid, common to both strains. Pale colours show the outcome of competition with the K12ΔarcA strain. Results are shown as means ± SEM (*N* ≥10). Data are available from FigShare at http://dx.doi.org/10.6084/m9.figshare.3199252.

Discrimination in transfer occurs if during the encounters between a donor and potential recipients the plasmids are transferred to cells of the donor’s kind more often than would be expected based on its frequency in the population. Discrimination of plasmid recipients could be based on differences in the initial recognition between cells or differences in plasmid establishment in recipient cells. To search for evidence of discrimination, we analyse two available datasets [11,12] that quantify plasmid conjugation rates among different pairs of natural isolates. Both studies measured conjugation rates for the multiresistant R1 plasmid, among 10 strains from the ECOR collection [11] or 9 other natural *Escherichia coli* strains [12]. In each dataset, we compute normalized donor ability for each pair of donor and recipient strains (see Materials and Methods), which corrects for basal differences in donor ability between strains. We find that transfer to self occurs at rates higher than average in 18 out of 19 cases (Fig 2A). Additionally, in 8 out of 19 cases, the highest rate of transfer is from a strain to itself. Overall, transfer to self is 7.3 times higher than average donor ability over all tested isolates (two tailed *t* test for difference from 0 for normalized donor ability to self, *p* = 0.0003). In a mixed population with many different strains, the high rates of transfer to self we describe here would translate into a biased transfer between cells sharing donation alleles. This apparent discrimination does not imply that the same locus is responsible for high donor ability and for discrimination, as multiple genes could be involved in discrimination. However, the signal we observe in Fig 2A suggests that alleles for high donor ability and for discrimination in transfer are linked in natural isolates sufficiently to lead to an effective discrimination at the donor ability locus.

We next experimentally investigate if discrimination may allow for the selection of host transfer genes. We use two widely studied *E*. *coli* strains, the K12 strain MG1655 [35] and the B strain REL606 [36], and the multiresistant R1-19 plasmid [37]. K12 and B strains bear different restriction-modification systems [9], potentially leading to discrimination in plasmid transfer [38,39]. We first measure the conjugation rate in a well-mixed environment between all four combinations of K12 and B as donor and recipient strains (Fig 2B, red bars). We find that K12 is generally a better donor, but also that K12 transfers R1-19 plasmid to itself at a 5-times higher rate than to B (Mann-Whitney Wilcoxon test, *p* = 0.003). Overall, the K12 strain is an example of good donor strain displaying discrimination for transfer, in comparison to the lower donor B strain. Moreover, R1-19 carriage leads to a 54% reduction in exponential growth rate for K12 strain, compared to a 1.6% reduction only for B strain (S1 Fig). To test if part of the costly effect R1-19 has on K12 is due to donor ability, we use a K12-derived strain with a deletion in the *arcA* gene, a gene known to affect transfer [40], and the repressed R1 plasmid, which transfers approximately 1,000-fold less than R1-19 [37,15]. The K12ΔarcA mutant transfers R1-19 plasmid at a strongly reduced rate to both itself and B (Fig 2B, grey bars), and R1-19 cost is reduced as well (6.4%, S1 Fig). Similarly, R1 plasmid imposes almost no cost to K12 growth (1.8%, S1 Fig). Both results suggest that most of R1-19 cost to K12 is due to its high transfer rate. We then test whether K12 discrimination in transfer can lead to biased transfer towards other K12 cells in a well-mixed population and subsequent selection of the better donor strain. We compete the K12 and B strains by mixing them equally in a well-mixed population, with a common proportion of cells from each strain initially bearing R1-19 plasmid. In the absence of antibiotic selection, the better donor K12 decreases in frequency (Fig 2C, dark blue), showing a lower basal fitness than B in those culture conditions. When antibiotic selection is applied at the end of the competition by plating the population on kanamycin (Kn)-containing medium, only Kn-resistant, plasmid-bearing cells grow. When all cells initially bear plasmids, selection does not favour the K12 strain (Fig 2C orange, 19% decrease in K12 frequency, two sided *t* test for difference from 0, *p* = 0.003). However, when only a fraction (2.5%) of both K12 and B cells initially bear R1-19 plasmid, providing opportunity for plasmid transfer, K12 is selected (19% increase in K12 frequency, two sided *t* test for difference from 0, *p* = 0.009). Finally, to confirm that this specific selection of donors is due to R1-19 transfer to K12 cells, we analyse the outcome of competition when the *arcA* gene is deleted from K12 and transfer is impaired. In the absence of antibiotic selection (Fig 2C, light blue), the *arcA* deletion does not affect K12 fitness when plasmids are absent or rare and increases K12 fitness when all cells bear plasmids (11% decrease for K12ΔarcA versus 25% decrease for K12, two-sided *t* test, *p* = 0.043), possibly because of the reduced plasmid cost for K12ΔarcA. With antibiotic selection, the specific selection of K12 when a fraction of cells bear plasmids disappears for K12ΔarcA (Fig 2C middle, yellow bars, two-sided *t* test, *p* = 2.10^−5^), demonstrating that K12 selection was due to plasmid transfer. Discrimination effectively biases antibiotic resistance transfer strongly enough so that the better donor K12 strain is selected for in the presence of antibiotics. Thus, when transferred plasmids are needed for growth, discrimination in transfer towards kind, at naturally appearing levels, can be sufficient to select for the better donor.

### Selection for Donor Ability in Structured Populations

**Fig 3:**
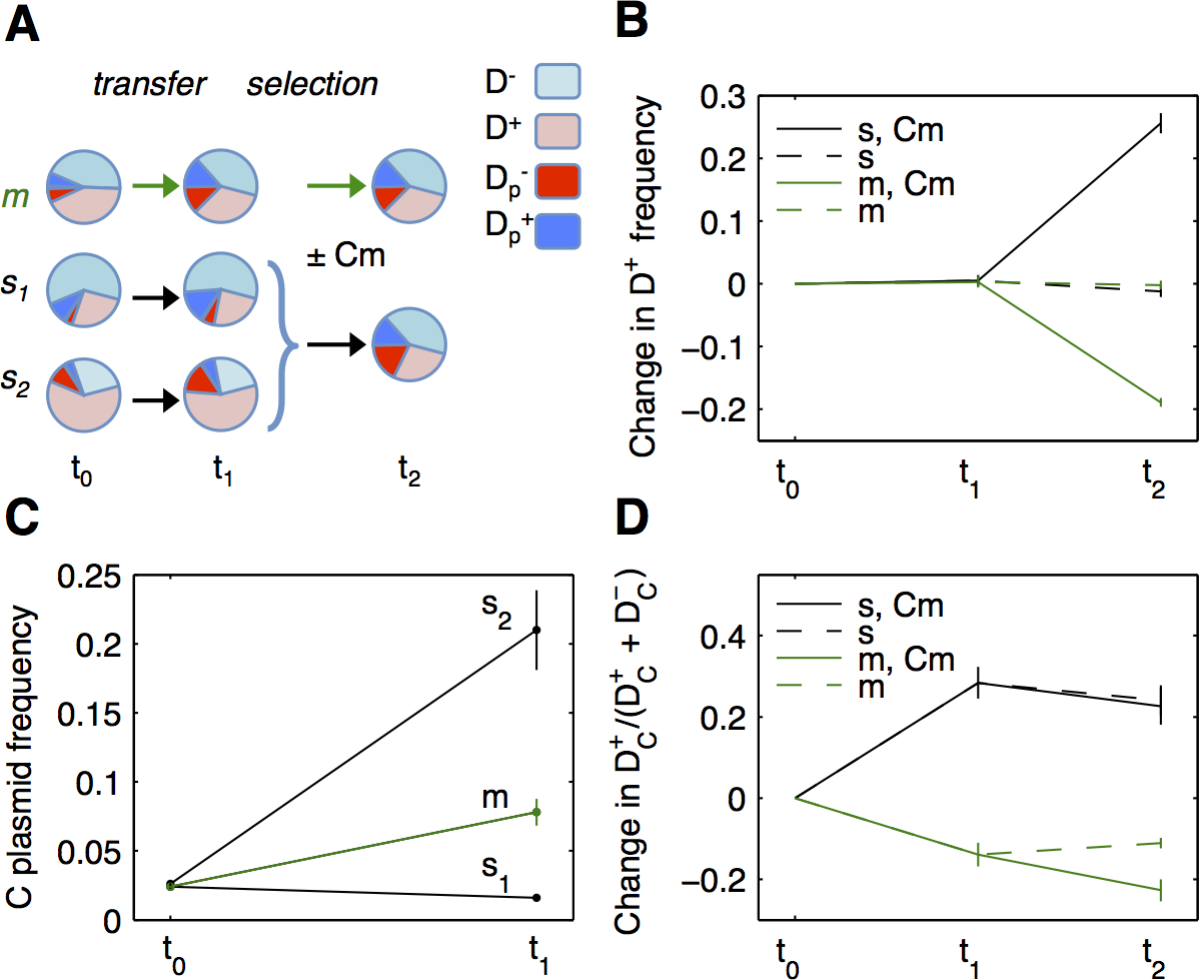
Selection of donor ability in structured populations. **A: Experimental setup.** D^+^ (good donor, red) and D^−^ (nondonor, blue) strains are competed. 2.5% of D^+^ and D^−^ cells initially carry C plasmids (bright colours), while 97.5% do not (pale colours). The population *m* is a single well-mixed population; metapopulation *s* consists of two subpopulations, *s*_*1*_ and *s*_*2*_, with initial D^+^/D^−^ ratios of 1/9 and 9/1. After growth and transfer (t_0_ to t_1_), subpopulations from *s* are pooled and cells are grown to saturation with or without antibiotic (Cm) selection (t_1_ to t_2_). The proportions of different cell types are represented schematically and do not correspond to actual numbers. **B: Selection of D**^**+**^ **strain.** The frequency of the good donor D^+^ is shown for *s* (black) and *m* (green) populations, with (plain lines) or without (dashed lines) Cm antibiotic during the selection phase. Good donors are only selected for in the *s* metapopulation, in the presence of antibiotic. **C: Plasmid dynamics.** Plasmid frequency in each population is shown for the transfer phase (from t_0_ to t_1_)_,_ in each of *m*, *s*_*1*_, and *s*_*2*_ populations. Plasmids spread mostly in the s_2_ subpopulation, enriched in the better donor, D^+^. **D: Transfer bias.** The proportion of C plasmids present in D^+^ strain, $D_{C}^{+}/(D_{C}^{+}+D_{C}^{-})$ is shown as a function of time for *s* and *m* populations (same colour scheme as in B panel). C plasmids get enriched in the better donor D^+^ strain during the transfer phase, for the structured population *s*. All results are shown as means ± SEM. (*N* ≥ 6). Data are available from FigShare at http://dx.doi.org/10.6084/m9.figshare.3199252.

A second possible reason for transfer bias is bacterial growth in structured populations, where donors interact preferentially with their kind. Next, we examine whether, in the absence of discrimination, structured populations can provide a sufficient bias in transfer to select for good donors. To analyse the effect of biased transfer in structured populations, we use a synthetic system with fluorescently tagged plasmids in which we can identify plasmid transfer between two strains. We adapted the system from the one we designed for an earlier study on interaction between conjugation and cooperation [6]. A helper plasmid F_HR_, that is nonmobile and thus behaves like a chromosomal allele, governs the host cell donor ability for a mobile plasmid C, which confers chloramphenicol (Cm) resistance. We compete (Fig 3A) two strains differing in their donor ability: the good donor D^+^ strain bears F_HR_ that transfers C plasmids, and the nondonor D^−^ strain does not (S2A Fig). After a transfer phase (t_0_ to t_1_), populations are grown with or without Cm during the selection phase (t_1_ to t_2_). We compare a single, well-mixed population (*m*), where D^+^ and D^−^ are mixed in equal proportions, to a structured metapopulation (*s*), consisting of two subpopulations that grow separately during the transfer phase, *s*_*1*_ and *s*_*2*_, founded respectively with a 10% and 90% proportion of D^+^ (leading to equal proportions of D^+^ and D^−^ at the metapopulation level). In this setup, the changes in the good donor frequency can be followed both within and among populations to evaluate the effect of population structure on donor selection.

D^+^ strain frequency does not change significantly in *m* or *s* populations during the transfer phase (Fig 3B left). D^+^ frequency then increases at t_2_ only for the structured *s* population grown in the presence of Cm (Fig 3B right, 26% increase from t_0_, Mann-Whitney Wilcoxon test, *p* = 0.004). It decreases for *m* population with Cm (19% decrease, two-sided *t* test for difference from 0, *p* = 3.10^−9^) and stays constant in the absence of Cm. The dynamics generally follows our predictions: D^+^ selection requires both population structure and plasmid selection. However, the expected cost of D^+^ during the transfer phase is not present at the population level. We next investigate in more detail both this cost and the selection of the good donor strain.

By looking at the dynamics of individual subpopulations during the transfer phase, we observed that D^+^ increases in frequency when prevalent (S3A Fig). We confirmed with an independent experiment that D^+^ fitness in competition with D^−^ linearly increases with D^+^ frequency (S3B Fig). This positive frequency-dependence for donor fitness could be due to lethal zygosis, a phenomenon known to damage recipients at high donor cell frequencies [41], which could be aggravated by the absence of entry exclusion in our strains [42]. In natural systems, entry exclusion may protect new transconjugants but would also make initial plasmid-bearing donors immune to lethal zygosis, probably leading to a similar frequency-dependence of fitness when most donor cells initially bear plasmids. In our system, frequency-dependence leads to no observable cost for D^+^ at the metapopulation level. At low frequencies, donor ability still has a cost, which is also observed as a decrease in the strain’s growth rate when growing in isolation (D^+^ versus D^−^, S2C Fig). Interestingly, donor cells grow significantly more slowly when they bear C plasmids, which is not the case for nondonor cells (D^+^_C_ versus D^−^_C_, S2C Fig), suggesting that donor ability cost is enhanced by the presence of transferable plasmids in the cell.

During the selection phase, good donors are selected only in the structured *s* population and only in the presence of Cm, meaning that donor selection requires both population structure during the transfer phase and subsequent antibiotic selection. We see that, as predicted by our model, biased transfer due to population structure promotes indirect selection of the donor strain. To better understand the factors affecting D^+^ selection, we proceed to analyse the dynamics of C plasmids (Fig 3C). During the transfer phase, plasmid frequency changes depend on the proportion of cells able to transfer. In the *s*_*1*_ population where D^+^ cells are few, plasmid frequency declines slightly. It increases mostly in the *s*_*2*_ population enriched in D^+^ strain. Increases are due to transfer, as the increase in plasmids present in D^−^ strain is due to plasmids that originate from D^+^ (as identified by fluorescence markers, see Materials and Methods and S4 Fig). We then follow the proportion of C plasmids that are present in D^+^ cells, as plasmid localization controls survival in the presence of antibiotics. During the transfer phase, the proportion of C plasmids present in D^+^ cells compared to D^−^ cells decreases in the well-mixed *m* population (13% decrease, Mann-Whitney Wilcoxon test, *p* = 0.004) but increases in the structured *s* population (28% increase, Mann-Whitney Wilcoxon test, *p* = 0.004) (Fig 3D). In the well-mixed population, the decrease is probably due to the strong fitness cost D^+^ cells incur specifically when they bear C plasmids (S2 Fig). The same cost also explains the subsequent decrease in D^+^ strain frequency under Cm selection in both populations. The enrichment of C plasmids in D^+^ cells depends on the population structure of the *s* population: total plasmid transfer is more prevalent in the *s*_*2*_ subpopulation, effectively biasing transfer towards D^+^ at the metapopulation level. These results experimentally confirm our models prediction: in the absence of discrimination mechanisms, donor ability for antibiotic resistance plasmids can be selected when population structure ensures preferential transfer to cells sharing donation alleles.

### Conditions Promoting Biased Transfer

So far, we have shown that both discrimination and population structuring can select for donor ability. However, we have always assumed and ensured that discrimination and population structure are present in the system. Here, we study how both phenomena can themselves emerge.

**Fig 4:**
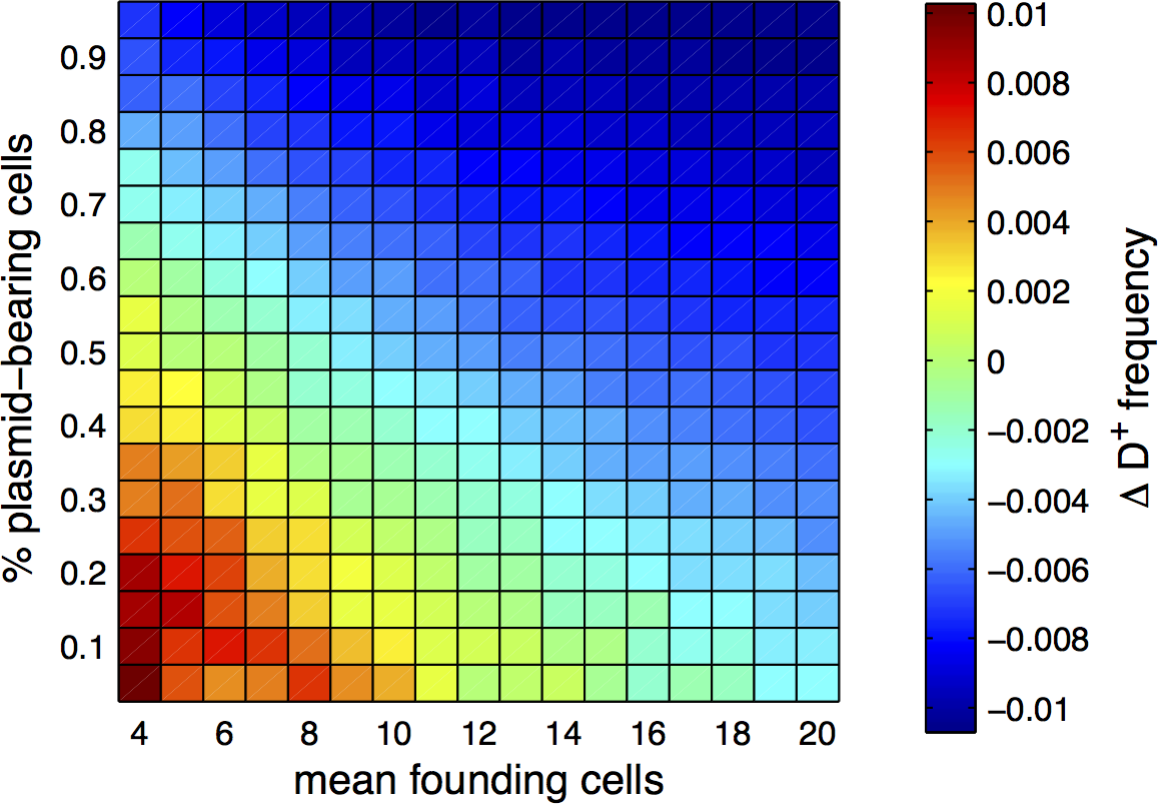
Selection of donor ability in a population structured by strong initial dilution. The simulated metapopulation consists of 192 subpopulations initiated from a strongly diluted mix of equal proportions of D^+^ and D^−^ cells, giving rise to a Poisson distribution of cell number across subpopulations for each cell type. The colour scale represents the change in D^+^ frequency from t_0_ to t_2_ averaged over 1,000 simulations, shown as a function of the initial proportion of plasmid-bearing cells and mean founding cell number per subpopulation after dilution. Data are available from FigShare at http://dx.doi.org/10.6084/m9.figshare.3199252.

When analysing effects of population structure, we imposed starting proportions of both the strains and the plasmids in order to dissect the dynamics of transfer. We continue by studying the outcome of competitions arising in more natural population structures using simulations. In the absence of a mechanism for discrimination of recipients, our model suggests that population structure may influence the selection of donor ability in two opposing ways (Eq 3). Efficient transfer is favoured by the coexistence of plasmid-bearing and plasmid-free cells within patches, while biased transfer towards kind is favoured by high relatedness at the donation locus. The selection of transfer thus requires preferential interactions between cells sharing donor ability alleles but also sufficient cell mixing that would favour the encounter between plasmid-bearing and plasmid-free cells. To study the effects of a natural population structure and the possibility that both conditions are met simultaneously, we simulate strong population dilution, which leads to stochastic founder cell numbers and genotype frequencies [6,33]. We follow the frequency of the donor D^+^ strain in a simulated metapopulation initiated from a strongly diluted mix of equal proportions of D^+^ and D^−^. With growth parameters based on our experimental results, we vary both the dilution factor applied to founding populations and the proportion of plasmid-bearing cells (Fig 4). The results exhibit a clear pattern: the donor strain is selected under the combination of strong initial dilution and low initial plasmid frequency. D^+^ selection in the presence of antibiotics is controlled primarily by the enrichment of plasmids in D^+^ at t_1_ (S5A Fig), which occurs when there is biased transfer towards D^+^ during the transfer phase. As predicted by Eq 3, biased transfer requires high relatedness at the donation locus but also effective transfer, which will be affected by dilution and initial plasmid prevalence. First, diluting down to low founding cell numbers provides sufficient variation in D^+^ frequencies among populations to ensure high relatedness (S5B Fig). Second, the contribution of transfer to plasmid abundance declines with increasing dilution and increasing plasmid initial abundance (S5C Fig). Transfer promotes plasmid invasion primarily when plasmid-bearing cells are initially scarce, because in those conditions, the majority of plasmid-bearing cells actually arise from transfer. Increasing dilution leads to the frequent absence of one of the cell types from each population, which in turn decreases the number of possible encounters between plasmid-bearing and plasmid-free cells. Selection for donor ability is strongest at high dilution because of high relatedness; however, dilution simultaneously limits transfer, which decreases the strength of selection compared to the optimal population structure studied in Fig 3. Overall, our simulations suggest that the conditions for selection of donor ability can be met in natural environments through limited dispersal alone, despite the trade-off between relatedness and transfer efficiency that arises from population structure.

**Fig 5:**
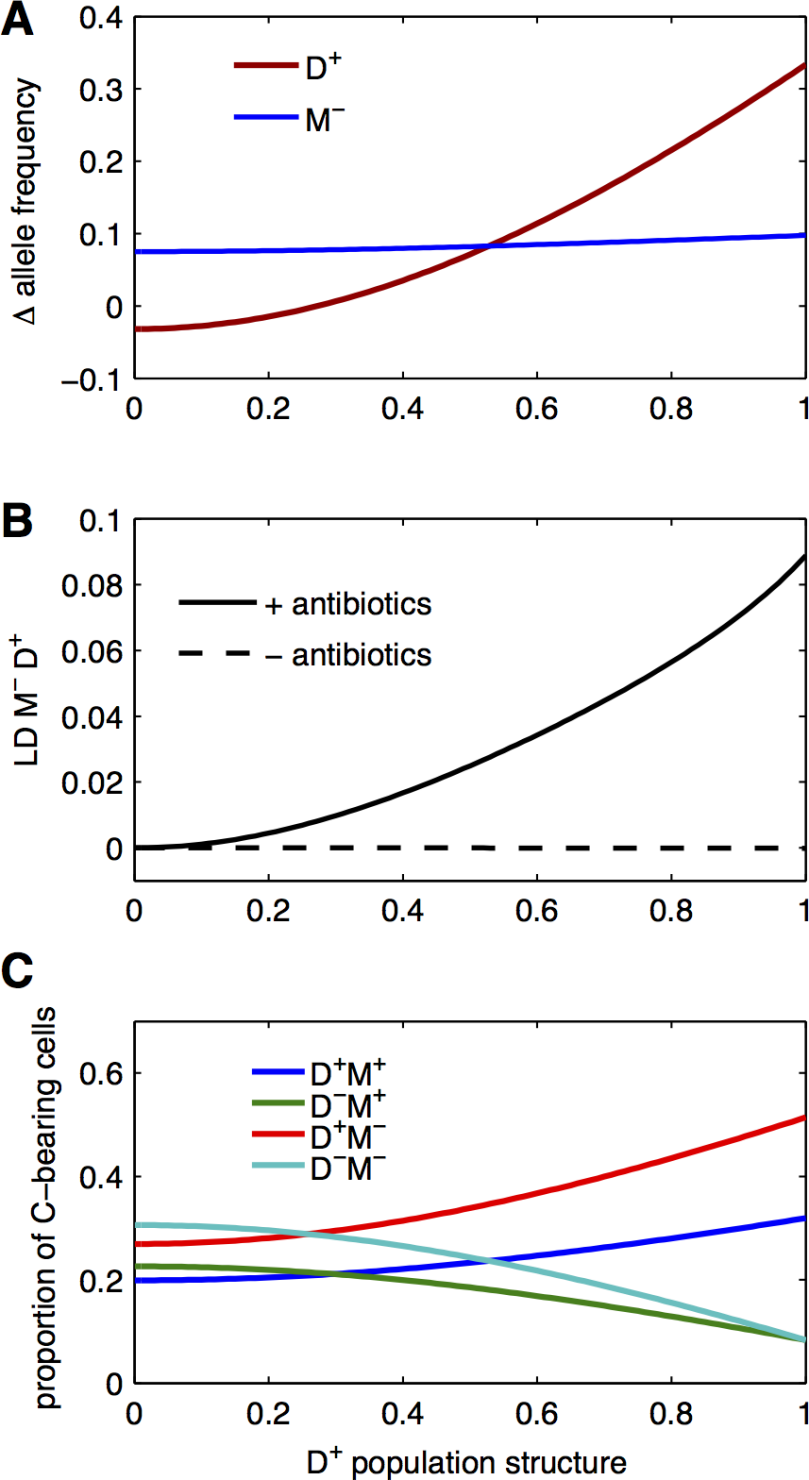
Emergence of linkage between donation and discrimination loci. **A: Selection of discrimination and donor ability.** The change in frequency of D^+^ (red) and M^−^ (blue) alleles after antibiotic selection is computed from simulations. The populations are analogous to structured populations *s* (Fig 3), but here we vary the strength of population structure (*x*-axis), expressed as the initial difference in D^+^ cell frequency between the two subpopulations. Initially, 2.5% of cells bear the antibiotic plasmid conferring antibiotic resistance. **B: Linkage between donation and discrimination alleles.** The linkage between D^+^ and M^-^ alleles is shown at the end of competition as a function of D^+^ population structure, calculated as before, in the absence (dashed line) and presence (bold line) of antibiotic selection that allows only plasmid-bearing cells to grow. **C: Plasmid transfer bias.** The proportion of each genotype among plasmid-bearing cells, at the end of competition is shown as a function of D^+^ population structure. With increasing population structuring, plasmids are progressively enriched in D^+^ M^−^ cells (red line). Data are available from FigShare at http://dx.doi.org/10.6084/m9.figshare.3199252.

We now focus on the association between discrimination mechanisms and plasmid transfer, which was apparent in natural isolates data (Fig 2A) and ask how this association itself could emerge. The effect on donor ability (Fig 2B and Fig 2C) suggests that a genotype bearing both discrimination and high donor ability alleles could be favoured by selection. We consider as an example the case of a strain inactivated at a chromosomal restriction-modification locus: such a mutant appearing in a wild type population will transfer plasmids preferentially to clonemates bearing the same allele, as unmodified plasmids transferred from this mutant will be degraded in a wild-type recipient cell [38,39]. The mutant allele with no modification is denoted by M^−^, and the wild type allele by M^+^, D^+^, and D^−^ stand for high and low donor ability, as before. We use simulations to follow the dynamics of modification and donation alleles, in populations of cells with no initial association between M^−^ and D^+^. In a well-mixed population (analogous to the one studied in Fig 2C), the M^−^ allele is selected for, but the D^+^ is not (Fig 5A, left). Discrimination in transfer by M^−^ cells leads to a reduced total transfer to M^+^ cells, and direct selection of M^−^ with comparatively higher recipient ability. D^+^ cells are outcompeted, as they do not receive more plasmids than D^−^ cells in the absence of association to M^−^ alleles. On the contrary, in a population where D^+^ cell frequency differs sufficiently among subpopulations, both M^−^ and D^+^ alleles are selected for (Fig 5A, right): similarly to the dynamics presented in Fig 3, population structure biases transfer towards D^+^ cells, allowing for D^+^ selection in the presence of antibiotics. Linkage between M^−^ and D^+^ alleles is also controlled by population structure (Fig 5B): with antibiotic selection, positive linkage appears when D^+^ cell frequency varies among subpopulations. This does not occur in the absence of antibiotics, suggesting that linkage is due to the specific selection of plasmid-bearing cells. With increasing D^+^ population structure, most plasmids end up in D^+^ M^−^ cells (Fig 5C) due to the combined effect of higher recipient ability and biased transfer by D^+^ cells. We conclude that the association between discrimination and transfer alleles can emerge simply through selection of plasmid-bearing cells, when population structure ensures that cells with high donor ability are favoured.

### Coselection of Host Donor Ability and Mutualistic Plasmids

Selective pressures acting on donor ability depend on the fitness effects of genes carried by the transmitted plasmids. Here, we focus on mutualistic antibiotic resistance plasmids, but parasitic and mutualistic plasmids can coexist in host populations, and hosts may not be able to evolve differential control of transfer based only on the accessory genes plasmids carry. Next, we consider the presence of parasitic plasmids N, which are similar to mutualistic plasmids C but do not confer benefits during the selection phase (see S2B Fig and Materials and Methods). In simulations, increase in the initial proportion of parasitic plasmids lead to a decrease in final frequency of the good donor strain (S6 Fig). We can conclude that the strength of selection for donor ability decreases when donors encounter a mix of mutualistic and parasitic plasmids, as they cannot distinguish between the two plasmid types. However, the benefits conferred by mutualistic plasmids could indirectly favour their association to the host.

**Fig 6:**
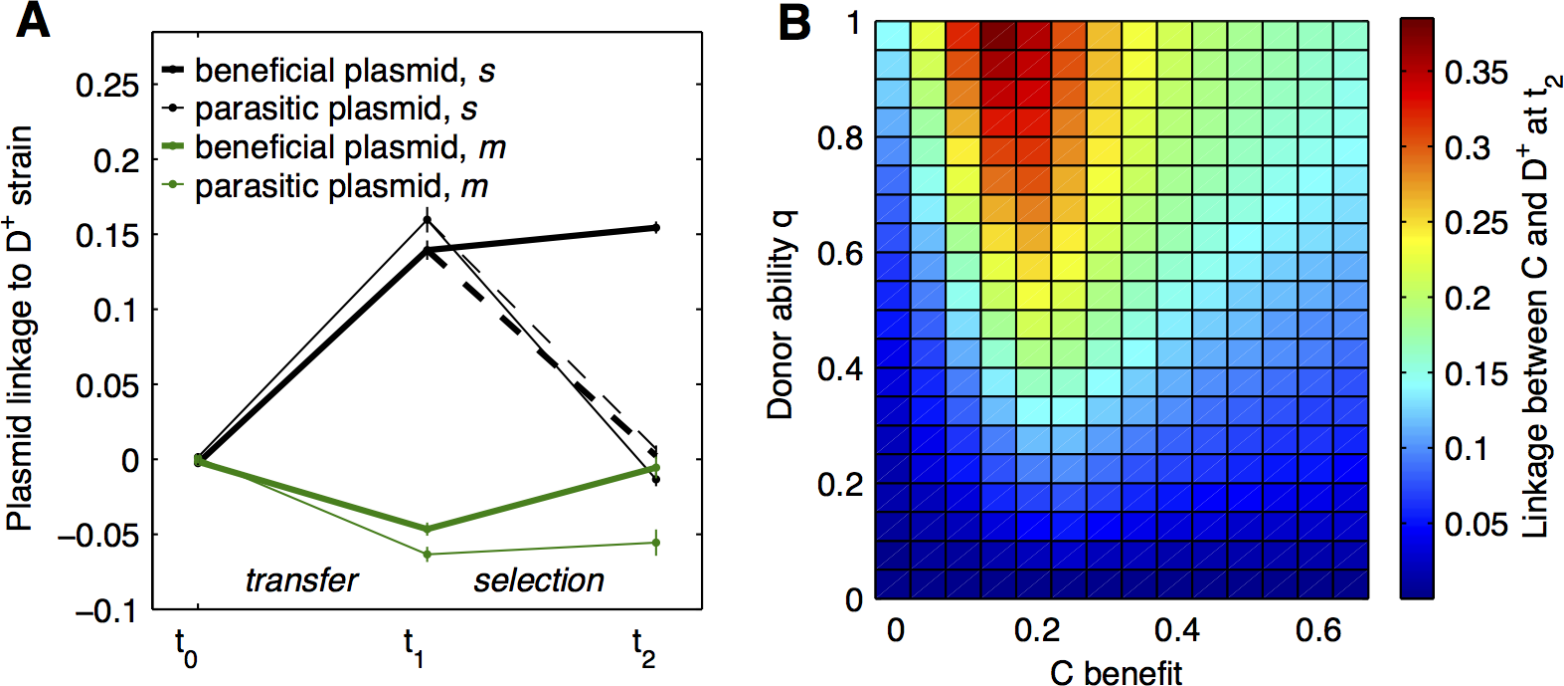
Coselection of donor ability and beneficial plasmids in the presence of parasitic plasmids. **A: Linkage of plasmids to D**^**+**^ **strain in experiments.** Linkage of beneficial Cm-resistant plasmids C (bold lines) and parasitic Cm-susceptible plasmids N (thin lines) to D^+^ strain is shown as a function of time, for *s* (black) and *m* (green) populations. 2.5% cells of each strain initially bear a plasmid, with equal proportion of C and N plasmids present. From t_1_ to t_2_, cells are grown in the presence of Cm (plain lines) or Kn in the case of *s* populations (dashed lines). Results are shown as means ± SEM. (*N* ≥ 7). **B: Effect of transfer and plasmid benefits on linkage.** Simulation data show the linkage of C plasmid to D^+^ strain at t_2_ as a function of D^+^ donor ability for the plasmid and the benefit C plasmid confers on growth during the selection phase. Data are available from FigShare at http://dx.doi.org/10.6084/m9.figshare.3199252.

To study the association between plasmids and hosts, we measure experimentally and with simulations the linkage disequilibrium between plasmids and the good donor allele in a metapopulation similar to the previous one (Fig 3) but now with a mix of C and N plasmids in equal proportions instead of only C plasmids, and with no initial association between plasmids and a specific strain (each plasmid is equally present in D^+^ and D^-^ strains). After the transfer phase, both C and N plasmids become significantly linked to the good donor D^+^ strain in the structured population, but not in the well-mixed population (Fig 6A, left). Moreover, after Cm selection, linkage to D^+^ slightly increases for C plasmids but decreases to zero for N plasmids in the structured population and remains at zero in the well-mixed population (Fig 6A, right, plain lines). Finally, this pattern relies on the specific selection of C-bearing cells: when selecting with Kn (an antibiotic to which both plasmids confer resistance) instead of Cm, linkage decreases to zero for both plasmids (dashed lines). Our experiments show that linkage between plasmids and good donor cells arises only when two specific conditions are met: (1) the population is structured, and (2) plasmids are beneficial.

To better understand the factors controlling the association, we independently vary D^+^ donor ability and C plasmid beneficial effect on the host strain fitness in our simulations. The linkage that appears at t_1_ between both plasmids and D^+^ strain increases with D^+^ donor ability, independently of subsequent plasmid benefits (S7A Fig). This linkage arises because transfer is biased towards D^+^ cells at the metapopulation level (as seen in Fig 3). In our experiments, the observed linkage is stronger for N plasmids because they are preferentially transferred. At t_2_, however, C plasmid benefits modify the linkage patterns. With increasing C benefits, C linkage to D^+^ increases (Fig 6B), and N linkage to D^+^ decreases (S7B Fig), when donor ability is sufficiently high. The specific association between C plasmids and the donor strain thus arises from the benefits provided by C plasmids to the host: antibiotics promote the selection of cells bearing C plasmids, which are mainly good donor cells because of previous transfer. Overall, this mechanism selects for cells simultaneously bearing both D^+^ and C alleles. Linkage between the good donor strain and beneficial plasmids arises without directly enforcing any association between the two, due to the combination of two effects: population structure biasing transfer towards good donor cells and the plasmids benefiting the host.

## Discussion

### Indirect Selection for Donor Ability

Our work investigates the evolution of host genes controlling the transfer of mutualistic MGEs such as those conferring antibiotic resistance. We focus on genes modulating plasmid donation, a property that, unlike plasmid reception, does not directly benefit the host. Earlier interpretations have described MGEs as a communal pool of genes conferring benefits at the population level [2,18]. We demonstrate here that donor ability, when it is costly to the host, is not selected directly. However, we do not need to invoke population-level benefits to explain why the host may promote MGE transfer. Instead, we show that host donor ability alleles can be selected indirectly when transfer increases their inclusive fitness (Fig 1). We then investigate further this qualitative result by measuring selection direction and strength in simulations as well as experiments using both natural and synthetic microbes, in situations close to ones that could be observed in nature.

### Mechanisms Promoting Biased Transfer

In the absence of discrimination, population structure is a simple mechanism ensuring that cells encounter preferentially neighbours of the same kind. Here, we demonstrate that in a synthetic biological system devoid of any mechanism for discrimination in transfer, population structure enables the selection of donor ability, biasing plasmid transfer prior to the selection of plasmid-bearing cells (Fig 3). Donor ability is not selected within well-mixed populations where donors do not interact preferentially with their kind, and good donors decline in frequency due to donor ability costs. However, donor ability is selected at a metapopulation scale, where population structure provides sufficient relatedness at the locus controlling donor ability. With simulations, we then show that populations with sufficient relatedness can arise simply through strong population dilution, despite the reduction in transfer due to fewer interactions between plasmid-bearing and plasmid-free cells (Fig 4).

Further, we experimentally demonstrate that differences in transfer rates between isolates, leading to effective discrimination in transfer, can also be sufficiently high to favour a strain with high donor ability (Fig 2). In natural isolates, we observe discrimination for the transfer of an antibiotic resistance plasmid. These results motivate future studies that would quantify the generality of discrimination by examining other plasmids and strains, as well as determine the underlying mechanisms. Discrimination can result from specific recognition during cell–cell contact [30] or even direct spread through the cytoplasm of clonemates in the case of bacterial chains [43]. Alternatively, discrimination can arise during the establishment of plasmids in recipient cells. In particular, plasmid transfer rate is greatly diminished when restriction-modification systems present in recipients differ from those in donor cells [9,38,39]. At a larger phylogenetic scale, a plasmid host range can be limited by its mechanisms of replication or transfer [44]. Even when plasmids are successfully transferred, they need not confer any fitness benefit, because genes beneficial in the initial donor may be suboptimal in a novel, unfamiliar host [45], favouring a donor strain over distant competitors in which the transferred accessory genes are not fully beneficial. Finally, discrimination may rely on quorum-sensing mechanisms regulating transfer [46], which can provide an indication of the local abundance of related cells.

Any of these mechanisms could lead to discrimination among transfer recipients, but they may not all be controlled by the same locus as donor ability. Discrimination by plasmid donors towards their kind necessitates genetic linkage between donation and discrimination alleles. The pattern observed for natural isolates (Fig 2A) suggests that a sufficient level of association does exist in nature, at least for the R1 plasmid. Moreover, this observation may be explained and maintained by the dynamics we describe in Fig 5, where linkage between discrimination and donor alleles emerges from their coselection in structured populations.

Biased transfer to kind can thus happen in host cells that differ from others at a single locus modulating donor ability in structured populations; the benefits of transfer then promote the emergence of discriminating genotypes through linkage with a second locus determining the specificity of transfer. Population structure plays a central role, allowing both the spread of donor alleles in the absence of discrimination mechanisms and the emergence of discrimination.

### Strength of Selection Acting on Donor Ability

As the selective pressures we describe here are indirect, they may be too weak to have a significant effect on the evolution of transfer rates. To examine this, we calculate selection coefficients acting on the donor allele in our experiments and simulations. The strength of selection observed for the discriminating strain K12 in competition with B is high (s = 0.35, S8 Fig). As the degree of discrimination displayed by K12 is close to the average one measured across natural isolates (Fig 2A), this result suggests that selection of donor strains that transfer preferentially to their kind may occur widely in nature, even in unstructured populations. In the structured populations we studied, the strength of selection depends on the details of population structure: when relatedness at the donor ability locus is high but plasmids are present in each strain in equal abundance, donors are again efficiently selected for in our experiments (s = 0.10, S9 Fig). When both parameters are controlled purely by initial dilution, they behave in opposing ways and selection is lower (S10 Fig, s = 0.0025 in the optimal case). In natural populations, selection arising through population structure might thus be weaker than the one due to discrimination in transfer and vary depending on the details of host and plasmid population dynamics. Still, bacteria are characterized by large population sizes, leading to estimates of effective population sizes around 10^7^ [47,48], which implies that mutations with selection coefficients larger than 10^−7^ can be selected for [49]. Thus, even in the presence of a trade-off between relatedness and transfer efficiency, selection acting on hosts can result in biologically significant changes in transfer rates.

### Plasmid Prevalence and the Selection of Donor Ability

In the long-term, continued selection for transfer requires that plasmids do not spread to fixation. As plasmid transfer itself increases plasmid prevalence in host populations, selection for donors will be progressively decreased with plasmid spread. However, many factors may contribute to maintaining plasmids at intermediate frequencies in bacterial populations. Accessory genes on plasmids are often beneficial in transient or local conditions [50,51] and could be repeatedly lost when they are not selected for. Plasmid-free segregants occur regularly and will rapidly invade populations when plasmids are costly. Other factors like the presence of bacteriophages can also lead to unstable dynamics, increasing plasmid loss [52]. Moreover, transfer is strongly regulated as a function of environmental conditions [8] and could be induced specifically in the conditions where plasmid-bearing cells are favoured. A striking case of such a scenario are mobile elements providing tetracycline resistance, whose transfer is induced by subinhibitory concentrations of tetracycline [53]: transfer occurs in conditions where mobile elements are likely to increase host fitness in the near future, as indicated by antibiotic gradients. Regulation of plasmid transfer will also modify the cost of transfer to the host. In our model, we assumed that plasmid-bearing cells experience a constitutive cost proportional to donor ability, leading to a higher cost to donor genotypes when plasmids are abundant. Transfer can be repressed when plasmid-free cells are likely to be rare [46], leading to the expression of transfer genes only when transfer efficiency is maximised. Finally, on a wider scale, cell migration between populations that experience different selection pressures for plasmid traits strongly increases the potential for horizontal transfer, as the immigration of plasmid-free cells in populations where plasmid traits are beneficial prevents plasmid fixation and allows sustained transfer [54].

Transferring plasmids increases the donor allele inclusive fitness because it enriches cells of the same kind with beneficial alleles. This phenomenon can be compared to the evolution of teaching in animals: teaching of adaptive information can be selected when teachers and pupils are related [55]. The difference between genetic information transfer in bacteria and cultural transmission is that beneficial genes are by default also transmitted vertically (together with the donor allele), making transfer ineffective if they are already prevalent in the population. Thus in order to be selected, horizontal transfer needs to improve transmission to kind compared to vertical transmission. Indeed, horizontal transfer is selected mostly when initially only few cells bear plasmids (Fig 4 and S5C Fig), as in these conditions it allows a more rapid and efficient spread than vertical transfer.

Interestingly, the phenomenon of lethal zygosis suggested by the positive frequency-dependence observed in our synthetic system (S3 Fig) [41,42] could act on the selection of donor ability in a complementary way, by selecting for donor genotypes when plasmids are prevalent. Transfer would then be a spiteful behaviour, in this case, not because of the indirect effects of transferred genes but due to the direct damage to recipient cells.

### Selection for Spiteful Transfer?

Bacteria frequently encounter parasitic MGE decreasing fitness. Eq 3 suggests that the transfer of parasitic elements could be selected if it can be preferentially directed towards cells of another kind. The spread of parasites has been suggested to be a typical case of spiteful behaviour, since the donors may be immune to the negative effects of their parasites [56]. Bacteriophages are a well-known example, where phage lysogeny ensures that most cells of the initial strain are protected from lysis and phages preferentially lyse the cells of a competing strain, at the same time ensuring phage spread [57]. Similar mechanisms are not yet known for plasmids. However, transfer to unrelated cells is well described in the case of the Ti plasmid of *Agrobacterium tumefasciens*, where the T-DNA is transferred to plant cells [58], and specific transfer and gene expression ensure that another species produces resources. Even with no specific targeting, suboptimal effects of transferred genes could render the plasmid harmful, damaging specifically unrelated recipients and effectively leading to spite.

### Host–Plasmid Coevolution Shaping the Evolution of Transfer

We conclude that the inclusive fitness benefits conferred by transferred plasmids can lead to indirect selection for host donor ability. Plasmid transfer rates thus can be shaped not only by their direct effects on plasmid fitness [7] but also by their indirect effects on host fitness. The direction and strength of selection acting on donor ability will depend on the potential for plasmid transfer, its bias towards kind, the fitness effects of plasmids present in the host population, and the costs of transfer. Thus, all these factors might, at least in part, determine both the strikingly large variability of transfer rates observed among bacterial isolates [11,12] and the existence of high donor ability strains.

Our findings have consequences in the context of the fight against the spread of antibiotic resistance, as the indirect selection of donor strains could promote widespread dissemination of antibiotic resistance. Treatments that decrease the spread of MGEs have already been considered, like male-specific phages that inhibit plasmid transfer but also kill preferentially the cells that actively transfer plasmids [59]. Our work suggests that, the same as for other cooperative behaviours [60], bacteria resistant to such treatments may evolve, but relatively slowly [61], which should be taken into account when aiming to diminish horizontal transfer [62].

More generally, our results underline the active role hosts may play in the evolution of transfer rates and the necessity to take bacterial social interactions into account when studying plasmid transfer. Plasmids themselves often bear public good genes involved in host sociality and interaction with neighbouring cells [5,20], and plasmid transfer promotes host public good production by modifying relatedness in structured populations [6]. The indirect benefits of added public good production may in turn further favour the hosts that are investing in transfer.

Finally, we show that biased transfer in structured populations combined with selection of plasmid-bearing cells promotes association between hosts with high donor ability, discrimination mechanisms (Fig 5), and beneficial plasmids (Fig 6). Donor ability can be selected in the absence of initial linkage with discrimination alleles or mutualistic plasmids, but selection itself creates linkage at the population level. This dynamic will alleviate the cost of parasitic plasmids and lead to a prevalence of donor strains associated with mobile, transiently beneficial plasmids. In the long term, the phenomenon could promote mutualistic coevolution between beneficial plasmids and strains that transmit them at high rates to their kind, in a way analogous to the evolution of mutualism between species. The benefits generated by mutualism can create an association between mutualistic partners [63], while the association itself favours further mutualism [64,65]. Plasmids would be a special case of mutualism, with a complex and important role of horizontal transmission, a mechanism that is generally expected to inhibit mutualism [66] but here actually benefits both partners. Social selection promotes host investment in plasmid transfer, increasing plasmid fitness but simultaneously promoting host association to mutualist plasmids. This will likely lead to complex social selective pressures acting on plasmids themselves and shape the mobile gene pool.

## Materials and Methods

### Strains and Plasmids

To test for discrimination, the better plasmid donor was the *Escherichia coli* K12 strain MG1655_red_, which is MG1655 [35] marked with the *td−Cherry* gene. The worse donor was the *E*. *coli* B strain REL606 [36]. To measure conjugation rates, two spontaneous mutants resistant to rifampicin (Rif^R^) for each strain MG1655 and REL606 were used as recipients. The plasmid used was the multiresistant R1-19 plasmid (that provides resistance to Cm, sulfonamides, ampicillin, Kn, streptomycin, and spectinomycin) [37]. The K12ΔarcA strain was MG1655_red_ transduced with the Keio collection arcA deletion mutant [67].

To test for transfer selection in structured populations, we used two synthetic strains, D^−^ and D^+^, and two associated plasmids, C and N. D^−^ strain is *E*. *coli* K12 MG1655. D^+^ strain is a derivative of MG1655 marked with the *td−Cherry* gene and bearing the helper plasmid F_HR_. F_HR_ is a variant of the F plasmid with low self-transfer and entry exclusion [6], which provides efficient mobilization of plasmids carrying F *oriT* sequence. N plasmids bear F *oriT* and an *aph* gene providing Kn resistance, while C plasmids additionally carry a *cat* gene providing Cm resistance. N and C plasmids express either YFP or GFP under control of the strong promoter P_R_. For selection experiments (Fig 3), D^−^ strain initially bears C-GFP plasmid, and D^+^ strain initially bears C-YFP plasmid, in order to identify the origin of C plasmid (see S2A Fig). For linkage experiments (Fig 6), C-YFP and N-GFP plasmids were used respectively as C and N plasmids (see S2B Fig).

### Strains and Plasmid Construction

F_HR_ and N-GFP plasmids and their construction are described in detail in [6] (where N-GFP was called the T^+^P^−^ plasmid). N-YFP was constructed by amplification of YFP sequence with primers AGCGACTCGAGGATAAATATCTAACACCGTGCGTGTTGAC and AGCACAAGCTTTTCCCGGGTCATTATTTGTATAG, then ligation of N-GFP plasmid and the PCR product after digestion with XhoI and HindIII. To construct C plasmids, the *cat* gene was amplified from pKD3 plasmid [68] with primers TACTAAGACGTCAGGAACTTCATTTAAATGGCG and TACTAGCTCGAGAAGAGGTTCCAACTTTCACC. The PCR product was ligated into the corresponding (GFP or YFP) N plasmid after digestion with AatII and XhoI.

The D^+^ and MG1655_red_ strains were constructed by integration of the *pRNA1-tdCherry* gene construction on pNDL32 plasmid obtained from Nathan Lord (Paulsson laboratory, Harvard Medical School). pNDL32 was transformed into MG1655 with selection on 100 μg/mL ampicillin, then streaked twice at 30°C on LB-agar (Luria-Bertani, BD Difco). Colonies were streaked overnight on LB-agar at 42°C, and plasmid loss was confirmed by checking that clones were ampicillin-sensitive. F_HR_ was finally added to D^+^ strain by conjugation. To construct K12ΔarcA, the Keio collection arcA deletion mutant was used for P1 transduction of MG1655 _red,_ then the *kan* resistance cassette was removed with pCP20 plasmid [68].

Spontaneous Rif^R^ mutants of MG1655_red_ and REL606 were obtained by plating overnight cultures on LB-agar with Rif (Sigma-Aldrich) at 100 μg/mL.

### Growth and Experiment Conditions

Experiments were conducted under well-mixed conditions with 5 mL medium in 50 mL tubes (Sarstedt). Exponential growth rates (S1 Fig and S2C Fig) were measured in a Tecan Infinite M200 reader on 100 μL cultures with 50 μL mineral oil (Sigma) in 96-well plates, after 100-fold dilution from stationary phase cultures.

#### Discrimination experiments

For the experiments focusing on discrimination (Fig 2B and Fig 2C), cells were grown at 37°C in M9 minimal medium (BD Difco), with 0.4% glucose and appropriate antibiotics for precultures (Rif, 100 μg/mL for Rif^R^ mutants; Kn, 50 μg/mL for R1-19 plasmid-bearing cells).

To measure conjugation rates (Fig 2B), stationary phase cultures of donor (plasmid-bearing) and recipient (Rif^R^) strains were washed to remove antibiotics. They were then diluted 1/50 into fresh M9 medium and grown separately at 37°C until the cultures reached an OD (600 nm) of 0.2. 500 μL aliquots of donor and recipient cultures were then mixed and incubated at 37°C for 15 min, limiting secondary transfer. When ΔarcA mutants were used as donors, the incubation time was extended to one hour to allow detection of rare transconjugants. Finally, mixes were plated at appropriate dilutions on LB-agar containing Rif (measuring recipients and transconjugants), Kn (measuring donors and transconjugants) and Rif + Kn (measuring transconjugants only). Conjugation rates were measured as $\gamma =\frac{T}{DRt}$ (mL.cell^-1^.h^-1^) where *T*, *D*, and *R* respectively indicate the density of transconjugants, donors, and recipients, and *t* indicates the incubation time.

For competition experiments (Fig 2C), stationary phase cultures of both strains were washed then mixed at a 50/50 ratio into a well-mixed population, with a varying proportion of plasmid-bearing cells for each strain, and grown from a 1/10 dilution (t_0_). Mixes were then further diluted 1/10 at 37°C into medium lacking antibiotics when they reached the OD (600 nm) of 1.2, and grown to stationary phase. Finally, they were plated at appropriate dilutions on LB-agar with or without Kn selection. Strain and plasmid frequencies were followed by identifying MG1655_red_ mCherry fluorescence from colonies with an Illumatool (Lightools) with 540 nm excitation and 590 nm emission filters.

#### Structured populations experiments

For the experiments focusing on structured populations (Fig 3 and Fig 6), cells were grown in Luria-Bertani (BD Difco) medium. Antibiotics used were Kn (Sigma-Aldrich) at 50 μg/mL for selection experiments and 10 μg/mL for linkage experiments; Cm (Sigma-Aldrich) at 30 μg/mL for selection experiments and 6.25 μg/mL for linkage experiments. Antibiotic concentration was lowered in linkage experiments in order for all cell types to grow to some extent, allowing more precise linkage measurements.

Stationary phase cultures of D^+^ and D^−^ strains were first washed and mixed at a 50/50 ratio (well-mixed population *m*), 10/90 ratio (*s*_*1*_ subpopulation), and 90/10 ratio (*s*_*2*_ subpopulations) of D^+^ versus D^−^. A 2.5% proportion of each strain was bearing C plasmids for selection experiments (Fig 3) and C or N plasmids for linkage experiments (1.25% cells bearing C and 1.25% cells bearing N, Fig 6). Cultures were grown from a 1/10 dilution (t_0_), then further diluted 1/10 at 37°C into medium lacking antibiotics when reaching the OD (600 nm) of 3. Cultures were then diluted 1/10 at 30°C, and grown until stationary phase (t_1_). Finally, subpopulations *s*_*1*_ and *s*_*2*_ from the structured population (*s*) were pooled, and all cultures were diluted 1/100 at 30°C with or without antibiotics for the selection phase of the experiments, until stationary phase (t_2_).

Cultures were analysed for strain and plasmid proportions by flow cytometry after fixation, as described in [6]. Data acquisition was performed on the Cochin Cytometry and Immunobiology Facility. For each sample, 100,000 cells were analysed using a BD LSR Fortessa cell analyzer (BD Biosciences). D^+^ strain was identified with RFP, plasmids with YFP and GFP fluorescence (S2 Fig).

### Data Analysis

#### Discrimination in transfer in natural isolates

The two datasets we analysed were Table 1 from [11] and Table 2 from [12]. In both datasets for each pair of donor and recipient strains, we computed a measure of donor ability normalized over all recipient strains tested in order to detect differences in conjugation rate to different recipients. Let *i* indicate the donor strain and *j* the recipient strain, we compute the mean donor ability per strain, *γ*_*i*_, and the standard deviation in the said donor ability, *σ*_*i*_, using logarithmic values for conjugation rates. The normalized donor ability for a given pair is $D_{ij}=\frac{\gamma_{ij}-\gamma_{i}}{\sigma_{i}}$

#### Measures of selection

Fitness of a strain *i* relative to a strain *j* between times 0 and 1 was measured as *W*_*ij*_ = *ln*(*N*_*i*,1_/*N*_*i*,0_)/*ln*(*N*_*j*,1_/*N*_*j*,0_), where *N* represents cell densities [36]. When the cell densities were not known, the increase in number for strain *i*, *N*_*i*,1_/*N*_*i*,0_, was approximated as *f_i,1_/f_i,0_* * *x*, where *f* is strain *i* frequency in the population and *x* is the population increase in number. Selection coefficients were then calculated as *s* = *W* − 1 [36].

#### Relatedness

Relatedness at a locus is calculated as the linear regression coefficient connecting an individual’s allele *g* with the alleles of its interactants at the same locus, *G* [69]. For the locus controlling donor ability, we consider that *g =* 1 if individual is D^+^ and 0 if it is D^−^, and *G* is the proportion of D^+^ in the subpopulation of a focal individual. Let *d*_*i*_ and *n*_*i*_ be respectively the number of D^+^ and number of bacteria within subpopulation *i*, and *d*_*tot*_ and *n*_*tot*_ be respectively the number of D^+^ and number of bacteria in the metapopulation. Then, when subpopulations are of the same size, the relatedness of D^+^ cells is calculated as follows:

$$\beta_{G,g}^{q}=(\sum_{i}\frac{d_{i}}{n_{i}}\frac{d_{i}}{d_{tot}}-\frac{d_{tot}}{n_{tot}})/(1-\frac{d_{tot}}{n_{tot}})$$

#### Linkage disequilibrium

Linkage disequilibrium quantifies the deviation from random association between alleles at different loci. Here, we apply the concept to the association between the discrimination allele M^−^ and the D^+^ allele (Fig 5) and to the association between a plasmid and the D^+^ allele (Fig 6). For two alleles X and Y, ${LD}_{X,Y}=\frac{F_{XY}{-F_{X}F}_{Y}}{\sqrt{F_{X}(1-F_{X})F_{Y}(1-F_{Y})}}$, where *Fxy* is the frequency of individual cells bearing both X and Y alleles, and *Fx* and *Fy* are respectively the frequencies of X- and Y-bearing cells in the metapopulation. Positive linkage indicates that X and Y are associated with each other more than what would be expected from their individual frequencies.

### Numerical Simulations

Our simulations mimic the experimental conditions of strain growth and plasmid transfer in the same way as described in our previous work [6]. Plasmid transfer follows a mass-action law: the number of transfer events is proportional to both donor and recipient cell densities in the local population. The probability coefficient is the transfer rate constant γ (mL.cell^-1^.h^-1^). Strains are characterized by their donor ability *q* that modulates effective transfer and leads to a proportional cost of donor ability for the donor cell *c*_*q*_. Similarly to our experiments, we model two steps: a transfer phase (from t_0_ to t_1_), then a selection phase, in conditions where the plasmid genes affect growth (from t_1_ to t_2_). The length of the transfer phase is set to 12 h after 100-fold initial dilution from carrying capacity, and growth for the selection phase is allowed for 36 h after a second 100-fold dilution. Equations governing changes in cell densities, presented below, are common to the two steps. *N*_*tot*_ is the total cell density.

#### Donor ability with no discrimination

In these simulations (Fig 4 and Fig 6), two incompatible plasmids can be present. C plasmid (encoding Cm resistance) promotes growth during the selection phase; N plasmid (parasitic plasmid, not encoding Cm resistance) does not. D^+^ cells have donor ability *q*, D^−^ cells have no donor ability. C plasmid-bearing cells are noted by D_C_, N plasmid-bearing cells are noted by D_N_, and plasmid-free cells are noted by D_∅_.

$$\frac{dD_{\emptyset }^{+}}{dt}=[(\psi -c_{q}q)\;D_{\emptyset }^{+}-q\;\gamma \;(D_{C}^{+}+D_{N}^{+})\;D_{\emptyset }^{+}]\;(1-\frac{N_{tot}}{K})$$

$$\frac{dD_{\emptyset }^{-}}{dt}=[\psi D_{\emptyset }^{-}-q\;\gamma (D_{C}^{+}+D_{N}^{+})\;D_{\emptyset }^{-}]\;(1-\frac{N_{tot}}{K})$$

$$\frac{dD_{C}^{+}}{dt}=[(\psi -c_{q}q+x_{C})\;D_{C}^{+}+q\;\gamma \;D_{C}^{+}\;D_{\emptyset }^{+}]\;(1-\frac{N_{tot}}{K})$$

$$\frac{dD_{N}^{+}}{dt}=[(\psi -c_{q}q+x_{N})\;D_{N}^{+}+q\;\gamma \;D_{N}^{+}\;D_{\emptyset }^{+}]\;(1-\frac{N_{tot}}{K})$$

$$\frac{dD_{C}^{-}}{dt}=[(\psi +x_{C})\;D_{C}^{-}+q\;\gamma \;D_{C}^{+}\;D_{\emptyset }^{-}]\;(1-\frac{N_{tot}}{K})$$

$$\frac{dD_{N}^{-}}{dt}=[(\psi +x_{N})\;D_{N}^{-}+q\;\gamma \;D_{N}^{+}\;D_{\emptyset }^{-}]\;(1-\frac{N_{tot}}{K})$$

#### Donor ability and discrimination in transfer

In these simulations (Fig 5), only the mutualistic C plasmid is present. In addition to the D locus controlling donor ability, the cells are characterized by the M locus controlling restriction-modification. M^+^ cells do transfer indiscriminately towards M^−^ and M^+^, whereas transfer from M^−^ towards M^+^ cells is reduced by a factor *m*. We assume that there is no direct cost of the M^−^ allele to the cells. To follow the experimental design used in Fig 2, the selection phase is not explicitly modelled here, but we consider a selection at the end of the transfer phase where only plasmid-bearing cells survive.

$$\frac{dM^{-}D_{\emptyset }^{+}}{dt}=[(\psi -c_{q}q)\;M^{-}D_{\emptyset }^{+}-q\;\gamma \;(M^{-}D_{C}^{+}+M^{+}D_{C}^{+})\;M^{-}D_{\emptyset }^{+}]\;(1-\frac{N_{tot}}{K})$$

$$\frac{dM^{-}D_{\emptyset }^{-}}{dt}=[\psi {M^{-}D}_{\emptyset }^{-}-q\;\gamma (M^{-}D_{C}^{+}+M^{+}D_{C}^{+})\;M^{-}D_{\emptyset }^{-}]\;(1-\frac{N_{tot}}{K})$$

$$\frac{dM^{+}D_{\emptyset }^{+}}{dt}=[(\psi -c_{q}q)\;M^{+}D_{\emptyset }^{+}-q\;\gamma \;(M^{-}D_{C}^{+}/m+M^{+}D_{C}^{+})\;M^{+}D_{\emptyset }^{+}]\;(1-\frac{N_{tot}}{K})$$

$$\frac{dM^{+}D_{\emptyset }^{-}}{dt}=[\psi M^{+}D_{\emptyset }^{-}-q\;\gamma (M^{-}D_{C}^{+}/m+M^{+}D_{C}^{+})\;M^{+}D_{\emptyset }^{-}]\;(1-\frac{N_{tot}}{K})$$

$$\frac{dM^{-}D_{C}^{+}}{dt}=[(\psi -c_{q}q+x_{C})M^{-}D_{C}^{+}+q\;\gamma \;(M^{-}D_{C}^{+}+M^{+}D_{C}^{+})\;M^{-}D_{C}^{+}]\;(1-\frac{N_{tot}}{K})$$

$$\frac{dM^{-}D_{C}^{-}}{dt}=[(\psi +x_{C})M^{-}D_{C}^{-}+q\;\gamma (M^{-}D_{C}^{+}+M^{+}D_{C}^{+})\;M^{-}D_{C}^{-}]\;(1-\frac{N_{tot}}{K})$$

$$\frac{dM^{+}D_{C}^{+}}{dt}=[(\psi -c_{q}q+x_{C})M^{+}D_{C}^{+}+q\;\gamma \;(M^{-}D_{C}^{+}/m+M^{+}D_{C}^{+})\;M^{+}D_{C}^{+}]\;(1-\frac{N_{tot}}{K})$$

$$\frac{dM^{+}D_{C}^{-}}{dt}=[(\psi +x_{C})M^{+}D_{C}^{-}+q\;\gamma (M^{-}D_{C}^{+}/m+M^{+}D_{C}^{+})\;M^{+}D_{C}^{-}]\;(1-\frac{N_{tot}}{K})$$

The default parameter values used in simulations are shown in Table 1 and were estimated from our experimental data (except for the cost of donor ability in Fig 4, see below):

**Table 1:** Default parameter values used in simulations. Parameters were generally based on our experimental measurements (see Materials and Methods for details and exceptions).

Parameter	Notation	Transfer phase	Selection phase (competition)	Selection phase (plasmid linkage)
basal growth rate	*ψ*	1.28 h^-1^	0.06 h^-1^	0.17 h^-1^
carrying capacity	*K*	4.10^9^ cells. mL^-1^	4.10^9^ cells. mL^-1^	4.10^9^ cells. mL^-1^
cost of donor ability	*c*_*q*_	0.05 (0.005 for Fig 4)	0.05 (0 for Fig 4)	0.05
C effect on growth	*x*_*C*_	−0.02	0.8	0.7
N effect on growth	*x*_*N*_	−0.02	−0.01	−0.03
transfer rate	*γ*	10^−9^ mL.cell^-1^. h^-1^	0	0
donor ability	*q*	0.7	0.7	0.7
discrimination	*m*	5		

#### Population structure and specific parameters

To study the effect of strong cell dilution (Fig 4), cells were distributed in 192 populations each of 10 μL, following a Poisson distribution of varying parameter λ depending on the strength of dilution and the proportion *p* of plasmid-bearing cells. With *n* being the mean total cell number (varying with the strength of dilution), λ was $n\times \frac{p}{2}$ for D^+^_C_ and D^−^_C_ cells and $n\times \frac{1-p}{2}$ for $D_{\emptyset }^{+}$ and $D_{\emptyset }^{-}$ cells. Because of the strong initial dilution, the transfer phase duration was set at 36 h and the selection phase duration at 60 h. The cost of donor ability c_q_ was divided by 10 during the transfer phase and set to 0 during the selection phase to prevent excessive fitness loss for donor cells, assuming a regulation preventing the expression of transfer genes when densities are low and transfer unlikely. For each parameter combination, the results were averaged over 1,000 replicate simulations. The high number of simulations was necessary because Poisson distribution produces high variation between replicates, especially with low founding cell numbers and small initial proportion of plasmid-bearing cells.

To study linkage between donor ability and discrimination (Fig 5), competitors and initial conditions were the same as the ones for Fig 3 experiments, with each cell type $\left(D_{\emptyset }^{+},\;D_{p}^{+}{,D}_{\emptyset }^{-}{,D}_{p}^{-}\right)$ having an equal proportion of cells bearing *M*^+^ and *M*^−^ alleles. Additionally, population structuring concerning the D^+^ allele was varied by founding subpopulations *s*_*1*_ and *s*_*2*_ with varying D^+^ to D^−^ proportion (maintaining a 0.5 total proportion in *s* population). With *d*_*1*_ and *d*_*2*_ being respectively D^+^ proportion in *s*_*1*_ and *s*_*2*_, we quantify the strength of population structuring by *d*_*2*_ *− d*_*1*_.

## Supporting Information

S1 Fig Effect of plasmid presence on the growth rate of *E*. *coli* strains used for discrimination experiments. Growth rates were measured in 96-well plates in M9 medium at 37°C, similarly to the conditions of the competition experiment. The maximal growth rate for each strain (A) and plasmid cost derived from the effect on growth rate (B) were computed as means ± SEM of at least three separate growth kinetics. With *r*_∅_ and *r*_*p*_ being respectively a strain's growth rate without and with plasmid, plasmid cost was calculated as (*r*_*p*_ − *r*_∅_)/*r*_∅_. Data are available from FigShare at http://dx.doi.org/10.6084/m9.figshare.3199252.
(TIFF)

S2 Fig Synthetic bacterial strains to study the selection of donor ability. The two competing strains differ in plasmid donor ability: D^+^ (red) bears F_HR_ plasmid that confers high donor ability (indicated by pili), D^−^ (white) does not bear F_HR_ and can receive plasmids but not transfer them. D^+^_C_ and D^−^_C_ cells bear plasmid C that codes for resistance to the antibiotics Cm (Cm^R^) and Kn (Kn^R^) and fluorescent proteins (plasmid background colour). D^+^_N_ and D^−^_N_ cells bear plasmids N that code for resistance to Kn (Kn^R^) and fluorescent proteins (plasmid background colour). C and N plasmids can be transferred by D^+^ cells (black arrows) to both D^−^ and D^+^ plasmid-free cells. Transfer can occur to cells already bearing plasmids, but this is rare in our experiments because the initial frequency of plasmid-bearing cells is low. In **A**, strains used for competition experiments (Fig 3) are shown: C plasmids initially in D^−^ express GFP, and C plasmids initially in D^+^ express YFP. In **B**, strains used for linkage experiments (Fig 6) are shown: C plasmids express YFP and N plasmids express GFP. In **C**, growth rates were measured in 96-well plates with conditions similar to the ones of the transfer phase (37°C, no antibiotics, blue) and the selection phase (30°C, Cm, red). Values are shown as means ± SEM of six separate growth kinetics. Data are available from FigShare at http://dx.doi.org/10.6084/m9.figshare.3199252.
(TIFF)

S3 Fig Within-population strain dynamics during the transfer phase. **A:** The change in frequency of the good donor strain D^+^ from t_0_ to t_1_, shown for *s*_*1*_, *m*, and *s*_*2*_ populations of the experiments presented in Fig 3, suggests that D^+^ strain fitness is frequency-dependent. Results are shown as means ± SEM (*N* ≥ 6). **B:** To confirm the existence of positive frequency-dependence, the change in frequency of D^+^ strain was measured in a separate experiment for various starting frequencies of D^+^, in the same conditions than the competition transfer phase (t_0_ to t_1_). To highlight the frequency-dependence of fitness, the relative fitness of D^+^ strain was calculated from D^+^ versus D^−^ frequency changes, considering a total 1,000-fold growth of cells due to successive dilutions, and is shown on the bottom graph. Each point represents a replicate experiment, with three points for each initial D^+^ frequency. Data are available from FigShare at http://dx.doi.org/10.6084/m9.figshare.3199252.
(TIFF)

S4 Fig Plasmid dynamics during the transfer phase. The change in frequency of D^−^ cells bearing plasmid C-YFP (yellow) and plasmid C-GFP (green) is shown from t_0_ to t_1_ for *s*_*1*_, *m*, and *s*_*2*_ populations of the experiments presented in Fig 3. By design, C-GFP plasmids are initially present in D^−^ only (see Materials and Methods) and decline in frequency as they are not transferred. C-YFP plasmids are initially present in D^+^ strain only and increase in frequency in D^−^ cells because of transfer from D^+^ cells. Data are available from FigShare at http://dx.doi.org/10.6084/m9.figshare.3199252.
(TIFF)

S5 Fig Parameters governing the selection of donor ability after strong initial dilution. Simulations are the same as the ones described in Fig 4. In **A**, the change in D^+^ frequency from t_0_ to t_2_ is shown as a function of the proportion of C plasmids present in D^+^ strain before the selection phase (at t_1_), each point being the mean of 1,000 replicates. D^+^ change in frequency correlates strongly with the enrichment of C plasmids in the strain during the transfer phase. The relatedness at the locus controlling donor ability at t_0_ (**B**) and the proportion of plasmid-bearing cells in the population at t_1_ that were not present at t_0_ (**C**) are shown by colour scales, as a function of initial plasmid-bearing cells proportion and the mean number of founding cells present in each population after dilution. Data are available from FigShare at http://dx.doi.org/10.6084/m9.figshare.3199252.
(TIFF)

S6 Fig Effect of the presence of parasitic plasmids on the selection of donor ability. The change in D^+^ frequency from t_0_ to t_2_ is shown from simulation data (see Materials and Methods) as a function of the initial proportion of plasmids that are parasitic plasmids N. Results are shown for different values of C benefits on growth (with high C benefits, the decrease in fitness with parasitic plasmids is apparent only for high proportions of the parasitic plasmid). The metapopulation is the same as the structured *s* metapopulation described in Fig 3, but now with a mix of N and C plasmids of total frequency 2.5% and other parameters based on linkage experiments measurements (Fig 6). Data are available from FigShare at http://dx.doi.org/10.6084/m9.figshare.3199252.
(TIFF)

S7 Fig Dynamics of plasmid linkage to D^+^ strain with transfer and selection. The metapopulation is the one described in Fig 6. Plasmid linkage to D^+^ is shown as a function of D^+^ donor ability and C plasmid benefit on growth (during the selection phase), for both plasmids at t_1_ (**A**), and for N plasmid at t_2_ (**B**). The linkage values in (A) are the same for C and N plasmids, as the plasmids do not affect growth differentially before the selection phase, so we represented them with a single panel. Data are available from FigShare at http://dx.doi.org/10.6084/m9.figshare.3199252.
(TIFF)

S8 Fig Selection coefficients for the K12 strain in competition with B. Data are from the discrimination experiment, presented in Fig 2C. Data are available from FigShare at http://dx.doi.org/10.6084/m9.figshare.3199252.
(TIFF)

S9 Fig Selection coefficient for the D^+^ strain in competition with D^−^ in a structured population. Data are from the structured population experiment, presented in Fig 3B. Data are available from FigShare at http://dx.doi.org/10.6084/m9.figshare.3199252.
(TIFF)

S10 Fig Selection coefficients for D^+^ strain in a population structured by strong dilution. Data are from the simulations presented in Fig 4. Data are available from FigShare at http://dx.doi.org/10.6084/m9.figshare.3199252.
(TIFF)

S1 Text Schematic model details and analysis. (DOCX)

## Abbreviations

Cm: chloramphenicol
HGT: horizontal gene transfer
Kn: kanamycin
MGE: mobile genetic element
OD: optical density
Rif: rifampicin
SEM: standard error of the mean
