# Supplementary figures and images for: Indirect Fitness Benefits Enable the Spread of Host Genes Promoting Costly Transfer of Beneficial Plasmids
Source: PLoS Biol. 2016 Jun 7;14(6):e1002478. doi: 10.1371/journal.pbio.1002478 (PMC4896427; doi:10.1371/journal.pbio.1002478)

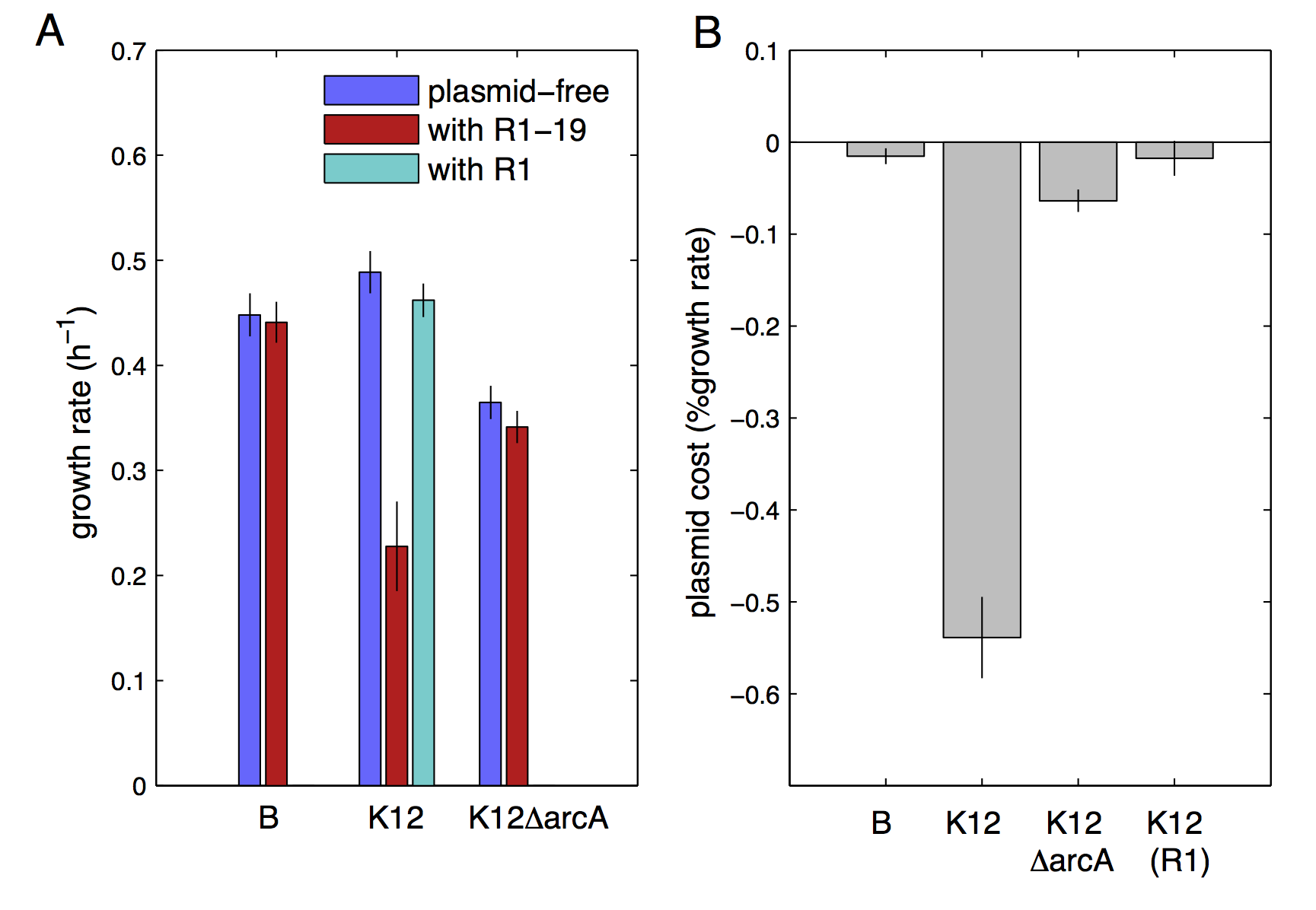

Supplement: S1 Fig — Growth rates were measured in 96-well plates in M9 medium at 37°C, similarly to the conditions of the competition experiment. The maximal growth rate for each strain (A) and plasmid cost derived from the effect on growth rate (B) were computed as means ± SEM of at least three separate growth kinetics. With r∅ and rp being respectively a strain's growth rate without and with plasmid, plasmid cost was calculated as (rp − r∅)/r∅. Data are available from FigShare at http://dx.doi.org/10.6084/m9.figshare.3199252. (TIFF) [file pbio.1002478.s001.tiff]

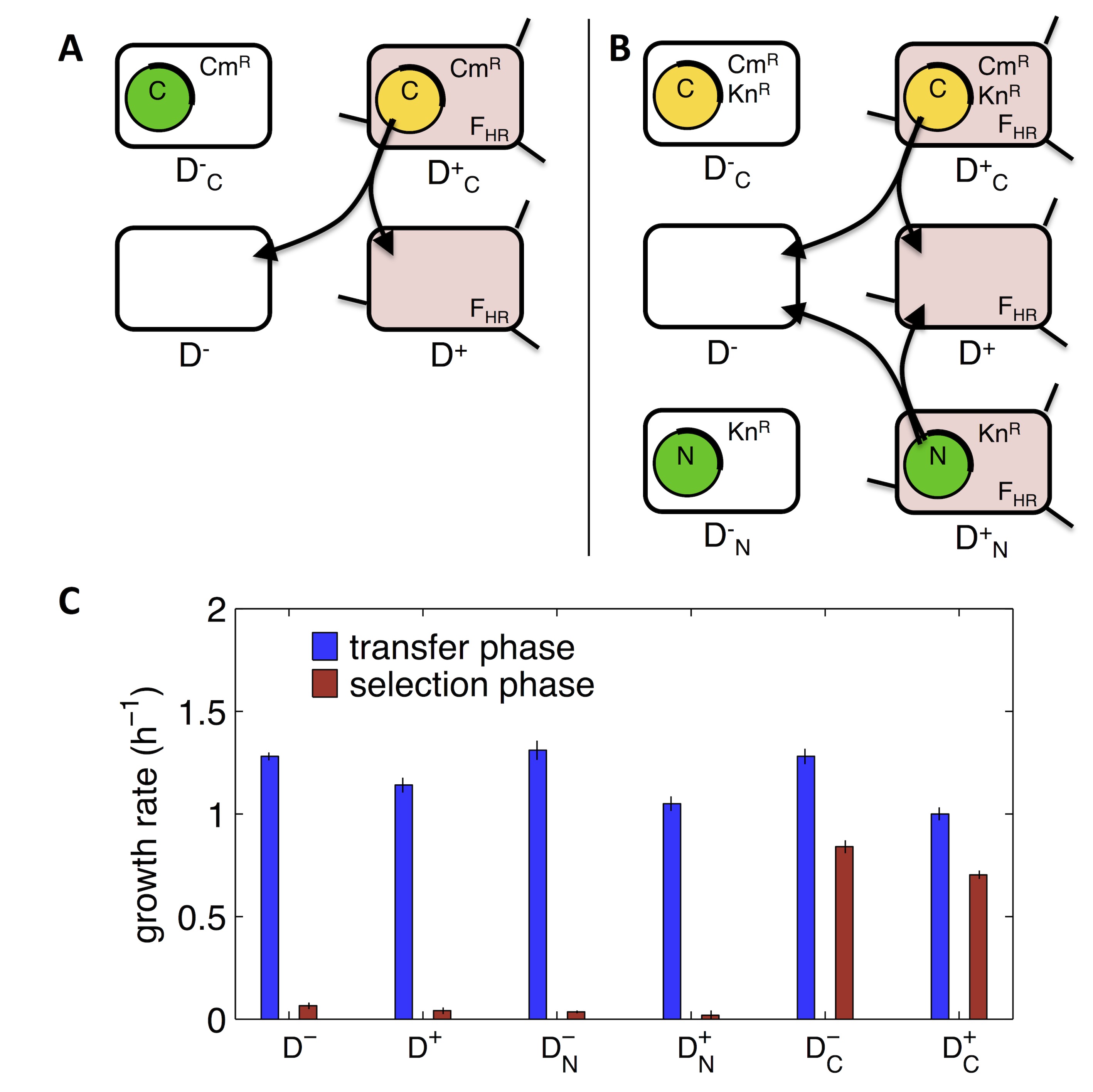

Supplement: S2 Fig — The two competing strains differ in plasmid donor ability: D+ (red) bears FHR plasmid that confers high donor ability (indicated by pili), D− (white) does not bear FHR and can receive plasmids but not transfer them. D+C and D−C cells bear plasmid C that codes for resistance to the antibiotics Cm (CmR) and Kn (KnR) and fluorescent proteins (plasmid background colour). D+N and D−N cells bear plasmids N that code for resistance to Kn (KnR) and fluorescent proteins (plasmid background colour). C and N plasmids can be transferred by D+ cells (black arrows) to both D− and D+ plasmid-free cells. Transfer can occur to cells already bearing plasmids, but this is rare in our experiments because the initial frequency of plasmid-bearing cells is low. In A, strains used for competition experiments (Fig 3) are shown: C plasmids initially in D− express GFP, and C plasmids initially in D+ express YFP. In B, strains used for linkage experiments (Fig 6) are shown: C plasmids express YFP and N plasmids express GFP. In C, growth rates were measured in 96-well plates with conditions similar to the ones of the transfer phase (37°C, no antibiotics, blue) and the selection phase (30°C, Cm, red). Values are shown as means ± SEM of six separate growth kinetics. Data are available from FigShare at http://dx.doi.org/10.6084/m9.figshare.3199252. (TIFF) [file pbio.1002478.s002.tiff]

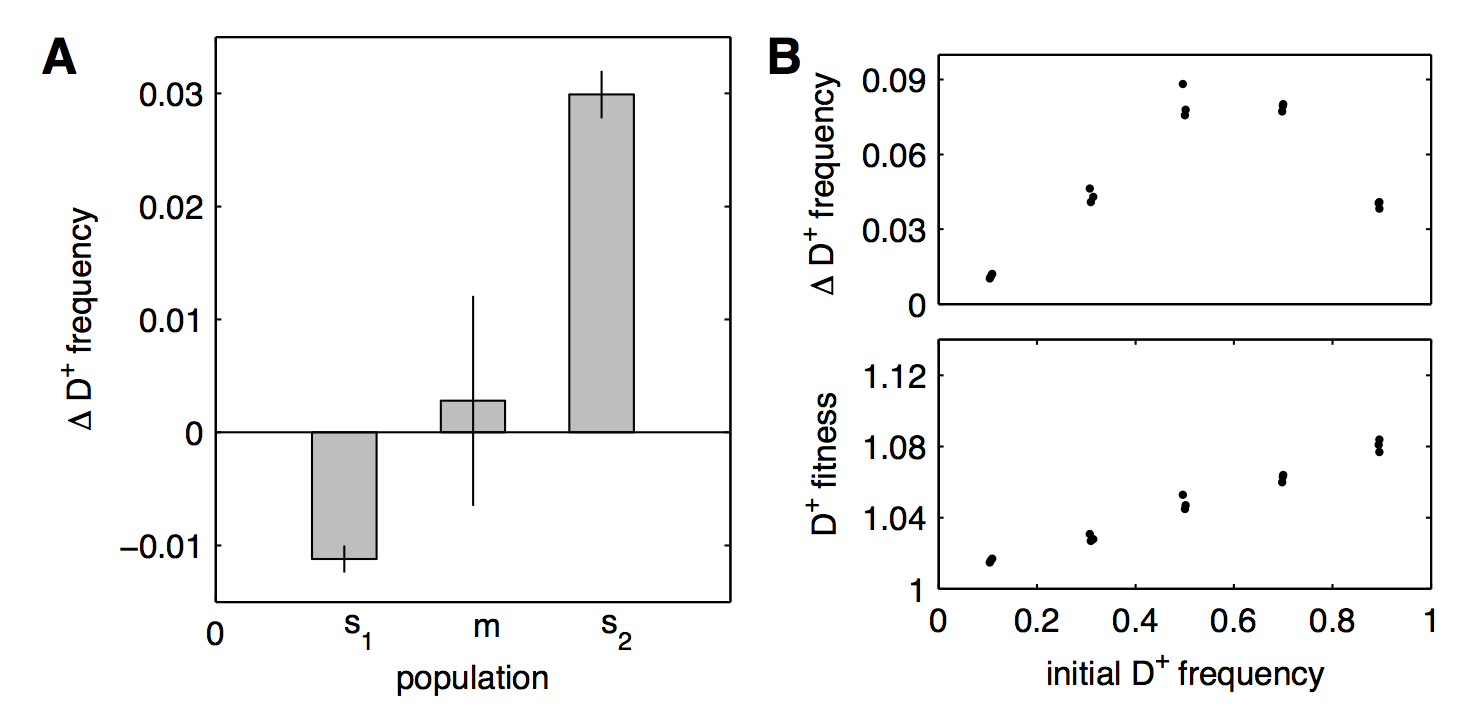

Supplement: S3 Fig — A: The change in frequency of the good donor strain D+ from t0 to t1, shown for s1, m, and s2 populations of the experiments presented in Fig 3, suggests that D+ strain fitness is frequency-dependent. Results are shown as means ± SEM (N ≥ 6). B: To confirm the existence of positive frequency-dependence, the change in frequency of D+ strain was measured in a separate experiment for various starting frequencies of D+, in the same conditions than the competition transfer phase (t0 to t1). To highlight the frequency-dependence of fitness, the relative fitness of D+ strain was calculated from D+ versus D− frequency changes, considering a total 1,000-fold growth of cells due to successive dilutions, and is shown on the bottom graph. Each point represents a replicate experiment, with three points for each initial D+ frequency. Data are available from FigShare at http://dx.doi.org/10.6084/m9.figshare.3199252. (TIFF) [file pbio.1002478.s003.tiff]

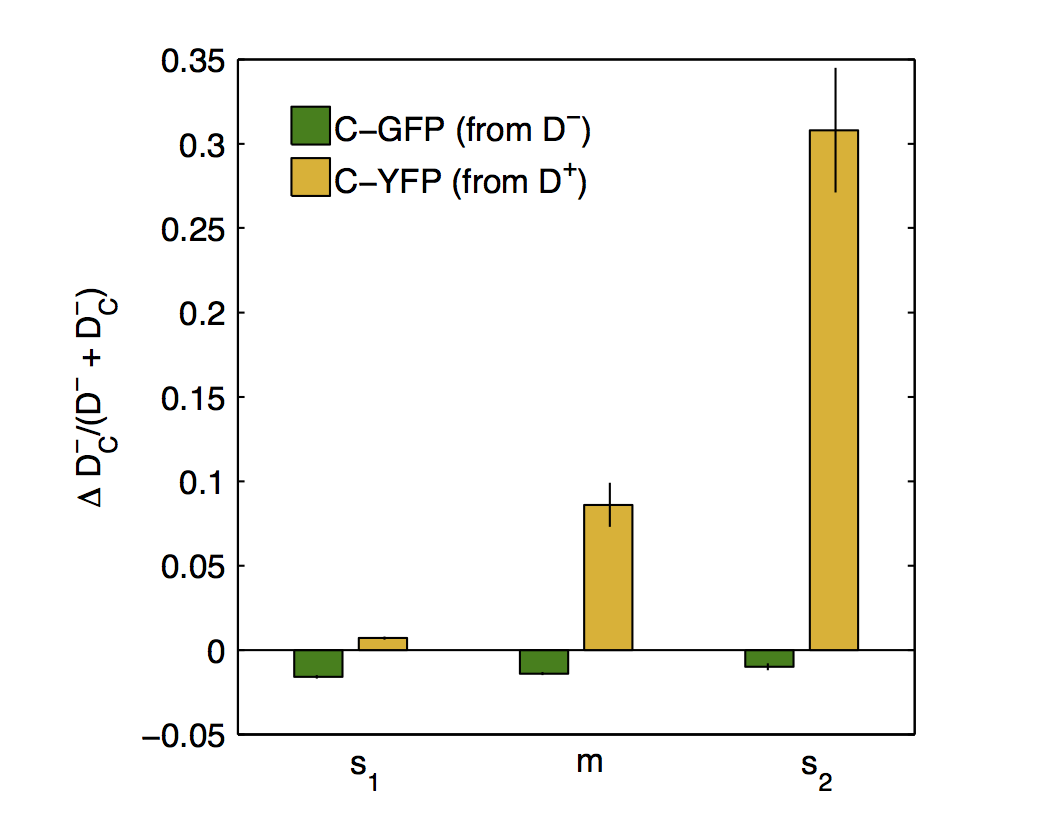

Supplement: S4 Fig — The change in frequency of D− cells bearing plasmid C-YFP (yellow) and plasmid C-GFP (green) is shown from t0 to t1 for s1, m, and s2 populations of the experiments presented in Fig 3. By design, C-GFP plasmids are initially present in D− only (see Materials and Methods) and decline in frequency as they are not transferred. C-YFP plasmids are initially present in D+ strain only and increase in frequency in D− cells because of transfer from D+ cells. Data are available from FigShare at http://dx.doi.org/10.6084/m9.figshare.3199252. (TIFF) [file pbio.1002478.s004.tiff]

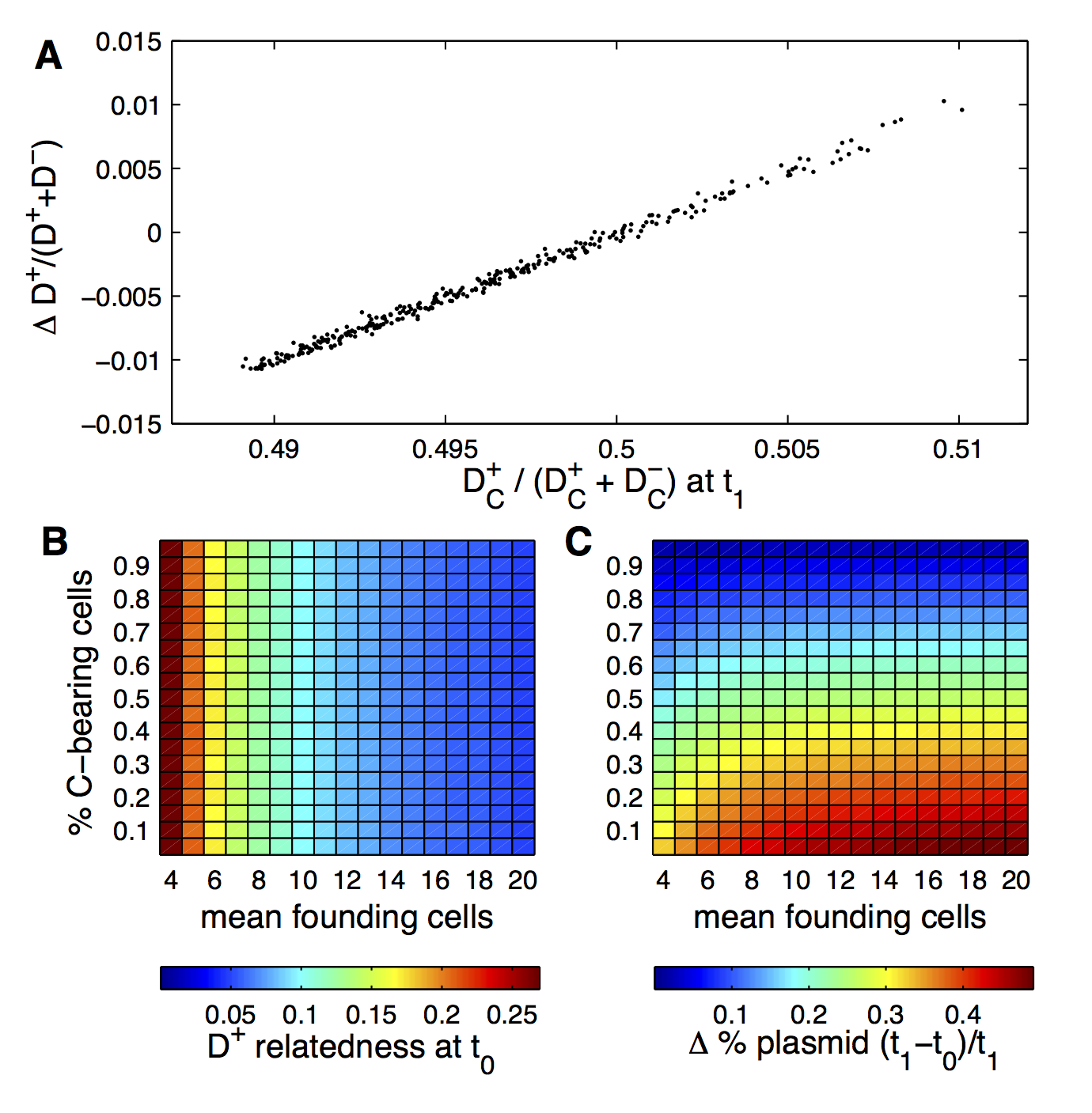

Supplement: S5 Fig — Simulations are the same as the ones described in Fig 4. In A, the change in D+ frequency from t0 to t2 is shown as a function of the proportion of C plasmids present in D+ strain before the selection phase (at t1), each point being the mean of 1,000 replicates. D+ change in frequency correlates strongly with the enrichment of C plasmids in the strain during the transfer phase. The relatedness at the locus controlling donor ability at t0 (B) and the proportion of plasmid-bearing cells in the population at t1 that were not present at t0 (C) are shown by colour scales, as a function of initial plasmid-bearing cells proportion and the mean number of founding cells present in each population after dilution. Data are available from FigShare at http://dx.doi.org/10.6084/m9.figshare.3199252. (TIFF) [file pbio.1002478.s005.tiff]

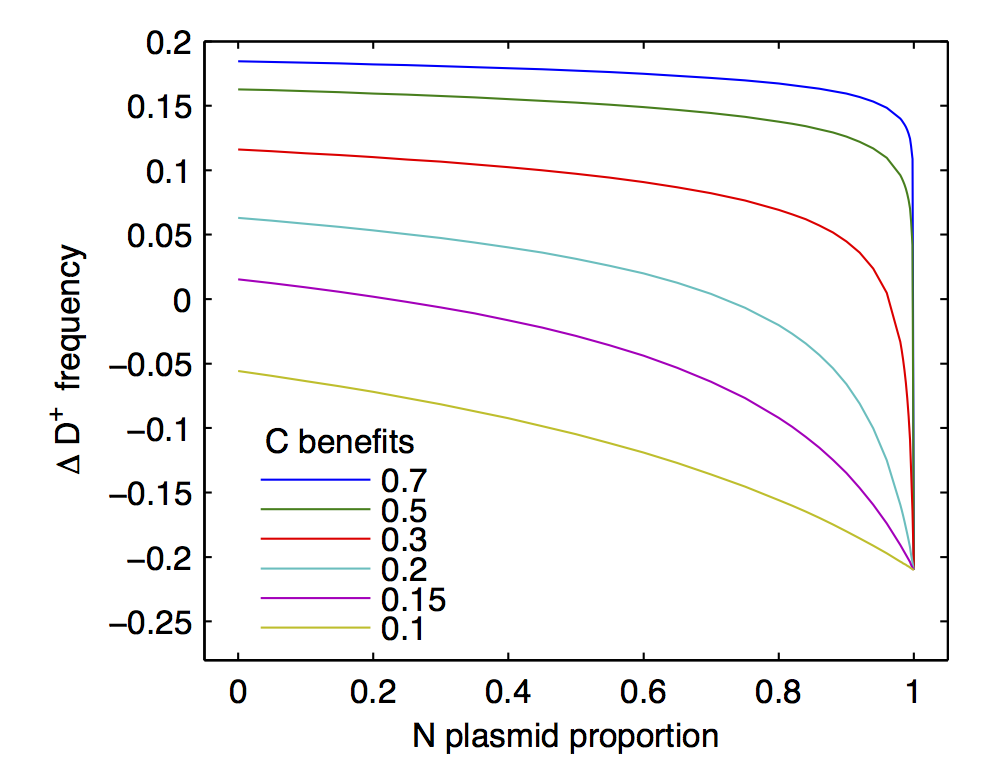

Supplement: S6 Fig — The change in D+ frequency from t0 to t2 is shown from simulation data (see Materials and Methods) as a function of the initial proportion of plasmids that are parasitic plasmids N. Results are shown for different values of C benefits on growth (with high C benefits, the decrease in fitness with parasitic plasmids is apparent only for high proportions of the parasitic plasmid). The metapopulation is the same as the structured s metapopulation described in Fig 3, but now with a mix of N and C plasmids of total frequency 2.5% and other parameters based on linkage experiments measurements (Fig 6). Data are available from FigShare at http://dx.doi.org/10.6084/m9.figshare.3199252. (TIFF) [file pbio.1002478.s006.tiff]

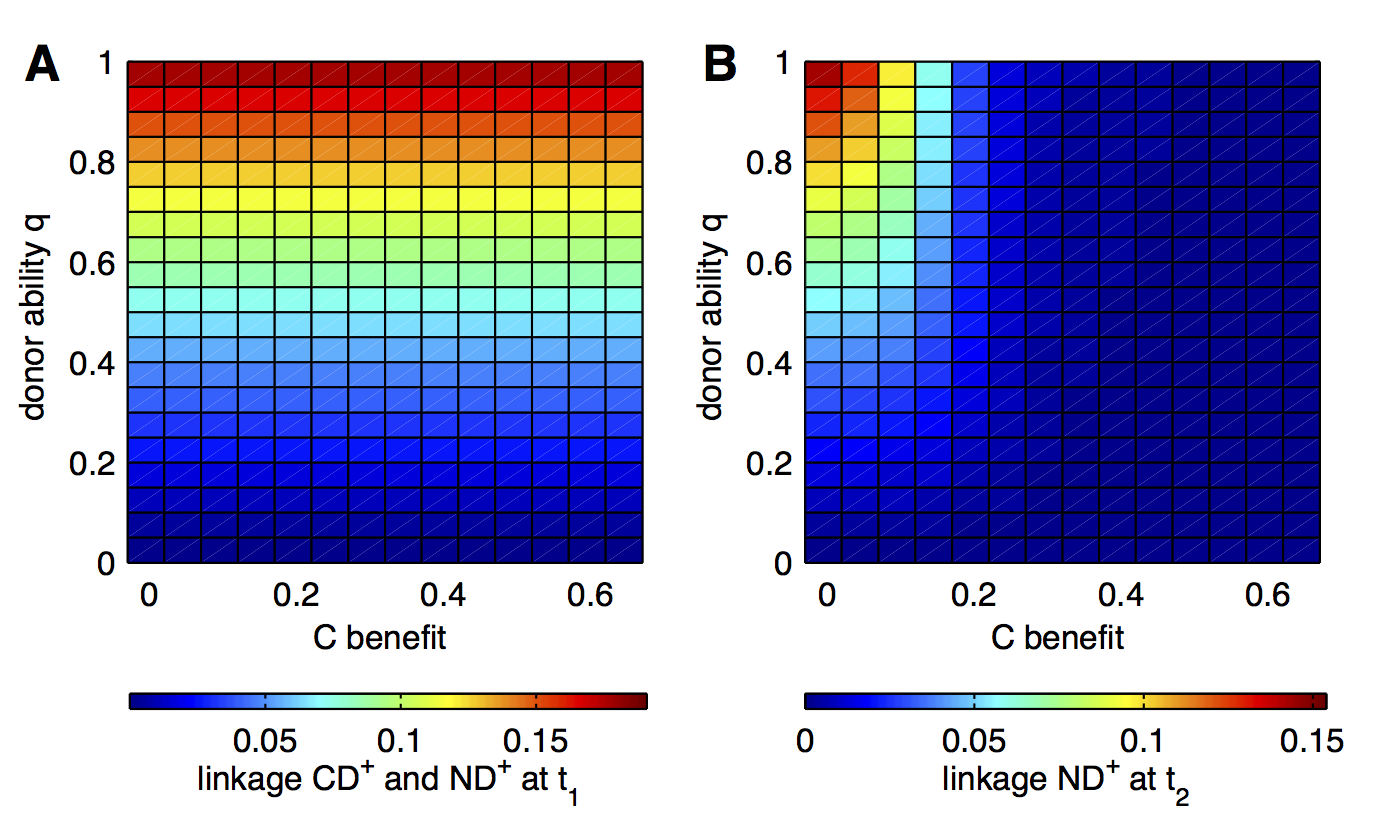

Supplement: S7 Fig — The metapopulation is the one described in Fig 6. Plasmid linkage to D+ is shown as a function of D+ donor ability and C plasmid benefit on growth (during the selection phase), for both plasmids at t1 (A), and for N plasmid at t2 (B). The linkage values in (A) are the same for C and N plasmids, as the plasmids do not affect growth differentially before the selection phase, so we represented them with a single panel. Data are available from FigShare at http://dx.doi.org/10.6084/m9.figshare.3199252. (TIFF) [file pbio.1002478.s007.tiff]

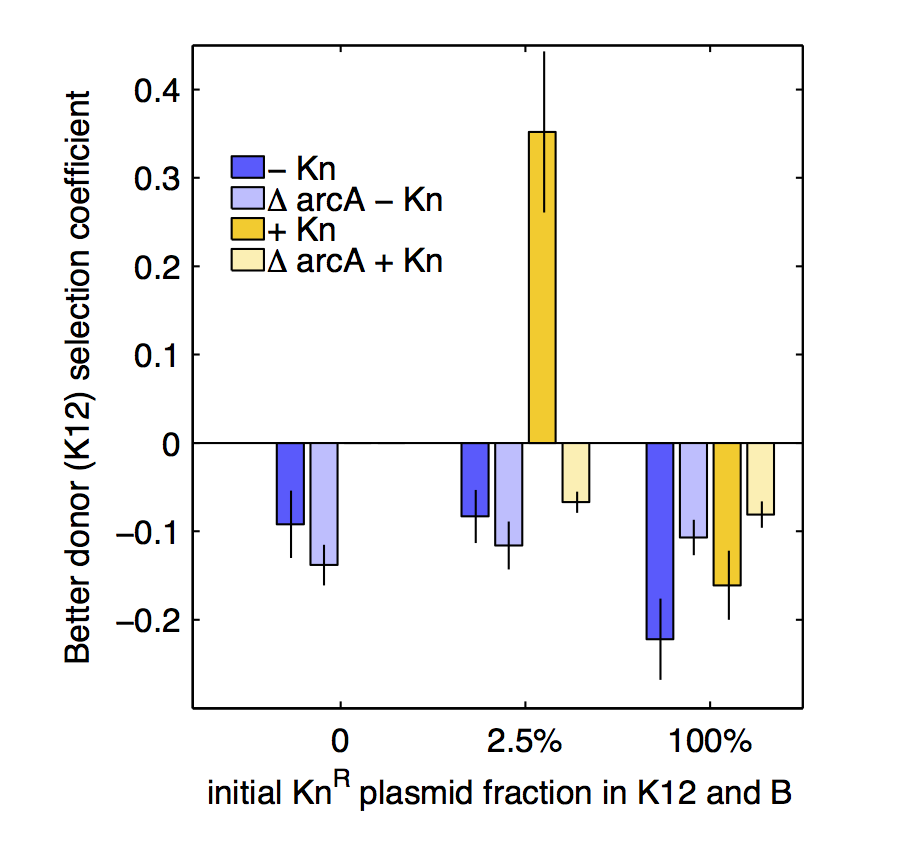

Supplement: S8 Fig — Data are from the discrimination experiment, presented in Fig 2C. Data are available from FigShare at http://dx.doi.org/10.6084/m9.figshare.3199252. (TIFF) [file pbio.1002478.s008.tiff]

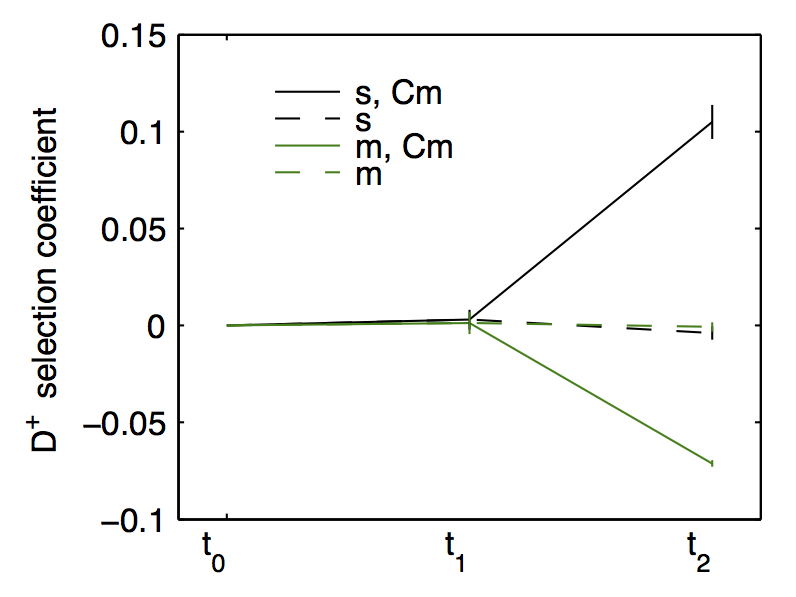

Supplement: S9 Fig — Data are from the structured population experiment, presented in Fig 3B. Data are available from FigShare at http://dx.doi.org/10.6084/m9.figshare.3199252. (TIFF) [file pbio.1002478.s009.tiff]

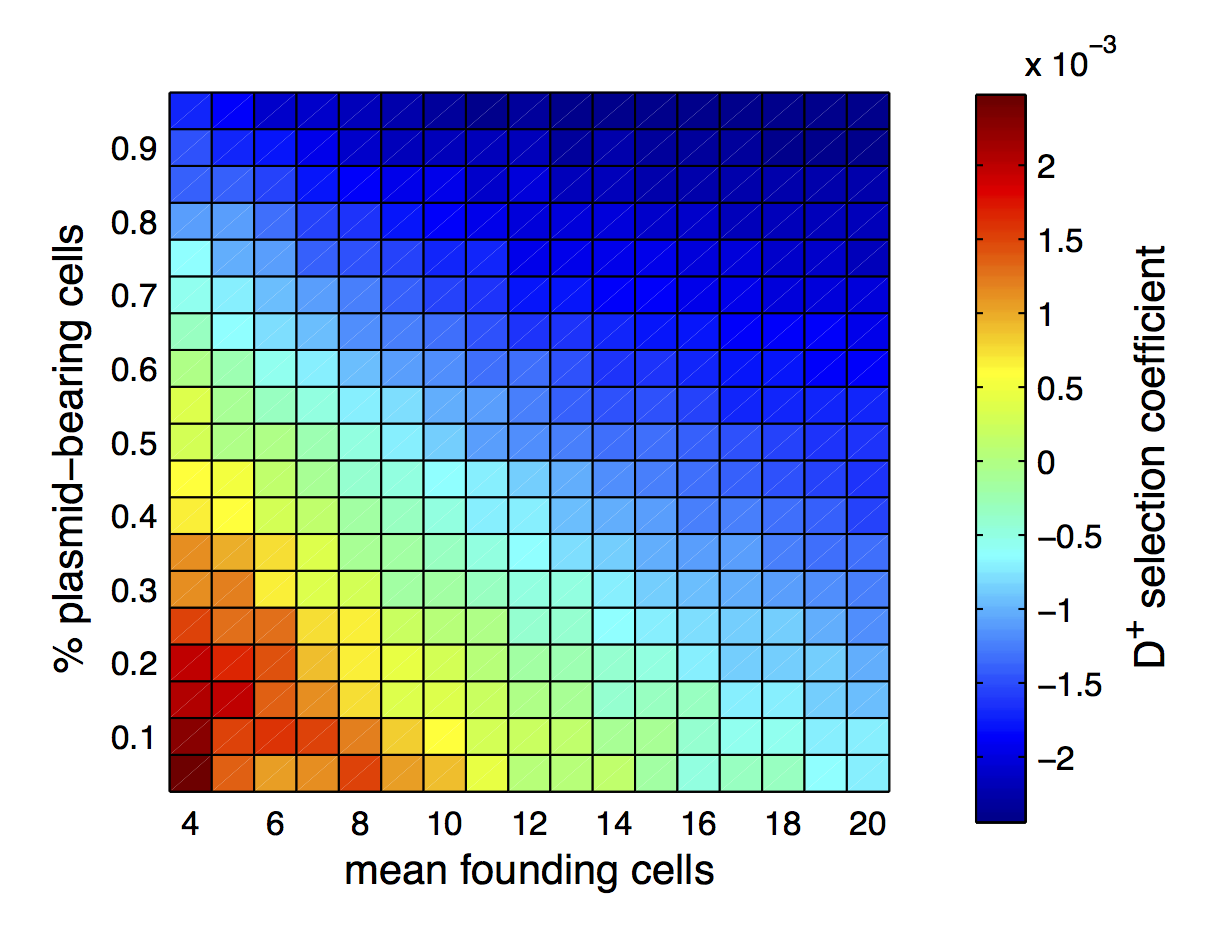

Supplement: S10 Fig — Data are from the simulations presented in Fig 4. Data are available from FigShare at http://dx.doi.org/10.6084/m9.figshare.3199252. (TIFF) [file pbio.1002478.s010.tiff]
