# Supplementary material for: Indirect Fitness Benefits Enable the Spread of Host Genes Promoting Costly Transfer of Beneficial Plasmids
Source: PLoS Biol. 2016 Jun 7;14(6):e1002478. doi: 10.1371/journal.pbio.1002478 (PMC4896427; doi:10.1371/journal.pbio.1002478)
Supplement: S1 Text — (DOCX) [file pbio.1002478.s011.docx]

**Text S1**

Our model focuses on the fitness of non mobile host genes modulating donor ability for a given plasmid. We consider that transfer is controlled by three factors: the probability of contact between plasmid-bearing and plasmid-free cells, the donor ability of plasmid-bearing cells, and the recipient ability of plasmid-free cells. Transfer may be variously successful for specific pairs of donor and recipient genotypes: in this model, this will translate into donor ability and recipient ability being related parameters.

*Model structure:*

As already described in the main text, we use a model that considers a population of bacteria structured in an infinite number of patches. An individual *i* in patch *j* is described by three properties, plasmid carriage $p_{ij}$ ($p_{ij}$= 1 for plasmid bearing cells and 0 for plasmid-free cells), donor ability $q_{ij}$ ($q_{ij}\in\left[ 0,1 \right])$and recipient ability $s_{ij}$ ($s_{ij}\in\left[ 0,1 \right])$. $q_{j}$, $s_{j}$ and $p_{j}$ are mean donor ability, recipient ability and plasmid frequency, respectively, in the patch *j*. Similarly, $q$, $s$ and $p$ are the mean donor ability, mean recipient ability and total frequency of plasmid-bearing cells in the entire population.

We model a simplified life cycle with non-overlapping patch generations, in which the following processes occur successively [25,26]:

*(a) Founding:* Patches are first colonized by founder cells sampled from the total initial population. We assume that plasmid and host traits are distributed independently in the starting population, so that the cell's donor ability $q_{ij}$ and recipient ability $s_{ij}$ are independent from its initial plasmid content $p_{ij}$. As a result of this, the plasmid costs or benefits are initially shared equally by all different host genotypes.

*(b) Reproduction:* Founder cells produce a large number of offspring by successive divisions, with vertical inheritance of plasmids. We assume that there is no plasmid loss, as rates of plasmid segregation are usually low.

*(c) Plasmid transfer:* Transfer occurs only within patches. For simplicity, we consider that transfer happens only once per life cycle (no secondary transfer) and only towards uninfected cells (no superinfection). Initially uninfected cells ($p_{ij}=0)$ become infected with a probability proportional to the patch level frequency of plasmid-bearing cells $p_{j}$, modulated by their own recipient ability $s_{ij}$ and by the average patch donor ability $q_{j}$. An individual cell *i* in patch *j* will thus be modified by transfer with the probability $(1-p_{ij})p_{j} q_{j} s_{ij}$. Its plasmid content after the transfer phase, noted $p_{ij}^{t}$, is therefore: $p_{ij}^{t}=p_{ij}+(1-p_{ij})p_{j} q_{j} s_{ij}$. Note that there is no explicit plasmid loss, a cell that carries the plasmid $(p_{ij}=1)$ necessarily remains the same after transfer, $p_{ij}^{t}=p_{ij}$.

*(d) Selection:* The fitness of a cell is function of two traits: plasmid content and donor ability. Plasmid presence has an effect $e_{p}$ on the host cell and we can express the plasmid effect on host fitness as $e_{p} p_{ij}^{t}$. Similarly, the cost of donor ability is $c_{q}$ ($c_{q}>0$) leading to an effect of transfer on host fitness that is proportional to donor ability, experienced only by cells bearing plasmids before transfer ($p_{ij}=1)$, and equal to $-{p_{ij} c}_{q} q_{ij}$. Donor ability is costly independently of actual transfer efficiency (the cost remaining unchanged even when, for instance, plasmid-free cells are not available in the patch, $p_{j}=1$, and transfer cannot happen). The cost we model is explicitly the effect of expressing the transfer machinery, which happens independently of the availability of plasmid-free cells or success of transfer.

*(e) Dispersal:* All cells from all patches disperse and form a panmictic pool of cells, from which the next generation of founder cells is sampled. As there is no population regulation in the previous phase, cells compete at the dispersal stage for representation in the next generation (global competition).

As already described in the main text, the fitness of an individual cell *i* in patch *j*, measured over the patch life cycle, is noted $W_{ij}.$ With $W_{0}$ being the basal host fitness, we can express the individual fitness as:

$W_{ij}={W_{0}+e}_{p} p_{ij}^{t}-{p_{ij} c}_{q}q_{ij}$

$={W_{0}+e}_{p} [p_{ij}+{(1-p_{ij})p_{j}q}_{j}s_{ij}]-{p_{ij} c}_{q}q_{ij}$ (1)

*Selection acting on donor ability:*

To understand selection on donor ability $q$, we apply the Price equation [27,28] to equation (1), under the assumption that $q_{ij}$ and $p_{ij}$ are independent, and $s_{ij}$ and $p_{ij}$ are independent. The covariance between $W_{ij}$ and $q_{ij}$, calculated across all individuals present in the population, is:

$$Cov\left( W_{ij},q_{ij} \right)= e_{p}Cov[{(1-p_{ij})p_{j}q}_{j}s_{ij}, q_{ij}] - c_{q} Cov({p_{ij} q}_{ij}{,q}_{ij})$$

$$= e_{p}\left( E_{ij}\left[ {\left( 1-p_{ij} \right)p_{j}q}_{j}{s_{ij}q}_{ij} \right]-E_{ij}\left[ {\left( 1-p_{ij} \right)p_{j}q}_{j}s_{ij} \right]E_{ij}\left[ q_{ij} \right] \right)$$

$- c_{q} (E_{ij}\left[ p_{ij}q_{ij}q_{ij} \right]-E_{ij}\left[ p_{ij}q_{ij} \right]E_{ij}\left[ q_{ij} \right]$)

$${=e}_{p}\left( E_{ij}\left[ (1-p_{ij})p_{j} \right]E_{ij}\left[ {{q_{j}s}_{ij}q}_{ij} \right]-E_{ij}\left[ (1-p_{ij})p_{j} \right]E_{ij}\left[ q_{j}s_{ij} \right]E_{ij}\left[ q_{ij} \right] \right)$$

$- c_{q} (E_{ij}\left[ p_{ij} \right]E_{ij}\left[ q_{ij}q_{ij} \right]-E_{ij}\left[ p_{ij} \right]E_{ij}\left[ q_{ij} \right]E_{ij}\left[ q_{ij} \right]$)

$$= e_{p}E_{ij}[{(1-p_{ij})p_{j}]{(E}_{ij}[q}_{j}{s_{ij}q}_{ij}]-{E_{ij}[q}_{j}s_{ij}]E_{ij}[q_{ij}]) - c_{q}E_{ij}\left[ p_{ij} \right] Cov(q_{ij},q_{ij})$$

$= e_{p}E_{j}\left[ p_{j}\left( 1-p_{j} \right) \right]Cov\left( q_{j}s_{ij}, q_{ij} \right)- c_{q} p Var(q_{ij})$

Dividing by $Var(q_{ij})$, we obtain the regression coefficient between fitness and donor ability, $\beta(W_{ij},q_{ij})$, which describes the effect of donor ability on fitness.

$\beta(W_{ij},q_{ij}) = e_{p} E_{j}[p_{j}(1-p_{j})]\beta(q_{j}s_{ij}, q_{ij}) - {p c}_{q}$ (2)

In the equation (2) just above, $\beta$ indicates regression coefficients and $E$ indicates expectations. The $E_{j}[p_{j}(1-p_{j})]$term describes the effect of patch composition on the efficiency of plasmid transfer: transfer events are more likely when both plasmid-bearing and plasmid-free cells are abundant within each patch. $E_{j}\left[ p_{j}\left( 1-p_{j} \right) \right]$ can also be expressed as $p\left( 1-p \right)-Var\left( p_{j} \right)$: transfer will be most efficient when both plasmid-bearing and plasmid-free cells are abundant at the population level (maximal when $p=0.5$), and $p_{j}$ deviates minimally from this frequency across patches.

The term $\beta(q_{j}s_{ij}, q_{ij})$is the regression coefficient between individual donor ability $q_{ij}$and the product of patch level donor ability $q_{j}$and recipient ability of individual cells$s_{ij}$. To better understand it, we can distinguish two extreme cases, based on variation in plasmid transfer parameters. In the first case, we consider a population in which there is no genetic variation in recipient ability among cells (transfer is solely controlled by the donor ability of plasmid-bearing cells and the probability of encountering recipient cells). Here, we can simplify the regression coefficient term, $\beta\left( q_{j}s_{ij}, q_{ij} \right)=s \beta\left( q_{j},q_{ij} \right)$. $\beta(q_{j},q_{ij})$ is the regression coefficient between patch-level and individual donor abilities, which corresponds to the relatedness at the locus controlling donor ability that arises from limited dispersal [22]. This relatedness is high when individual cells interact with cells sharing the same allele for donor ability within a patch. Alternatively, we consider a second case in which there is no variation in average donor ability among patches (all *q_j_* are equal, a cell's donor ability is not correlated to the one of surrounding cells). We can similarly simplify the regression coefficient, $\beta\left( q_{j}s_{ij}, q_{ij} \right)=q \beta\left( s_{ij},q_{ij} \right)$. $\beta(q_{j},q_{ij})$ is the regression coefficient between individual donor and recipient abilities. High $\beta\left( s_{ij},q_{ij} \right)$ means that genotypes with high donor ability also have higher recipient ability than average. This translates into discrimination in transfer by the cells with higher donor ability, as transfer from these cells will be biased to the neighboring cells sharing high donor ability. The two extreme cases we just considered demonstrate that $\beta\left( q_{j}s_{ij}, q_{ij} \right)$is effectively a summary relatedness parameter, combining population structure and specificity in transfer – it determines how much plasmids are transferred to recipient cells sharing alleles for high donor ability.

Finally, $p$ is the average, population level frequency of plasmid-bearing cells, leading to $p c_{q}$ as the average cost of transfer for the donor genotype: having high donor ability is costly to the proportion of cells that bear plasmids and invest into transfer.

With that, we have examined and explained all the terms in Equation (2), which leads to a variant of Hamilton’s rule explaining the maintenance of a cooperative behavior (Equation 3), as we detailed in the main text.

*Alternative mechanisms leading to discrimination in transfer:*

In the model presented here, discrimination arises from a positive correlation between donor ability $q_{\mathrm{ij}}$ (towards all recipient genotypes) and recipient ability $s_{\mathrm{ij}}$ (from all donor genotypes). This correlation effectively leads to discrimination, defined as higher transfer towards a given genotype than expected from its frequency in the population*.*

Another way to obtain discrimination in a well-mixed population, not explicitly modeled here, is an interaction between donor and recipient genotypes, such that the donor transfers plasmids preferentially to a specific recipient genotype, in a way that is not explained only by the recipient's average recipient ability. For instance, transfer will happen preferentially between cells that share restriction-modification systems. In the natural isolates that we analyzed, transfer is significantly higher from donors towards their kind than towards other strains (Fig 2A), however mean donor ability and recipient ability are not correlated [11,12]. In our own experimental data (Fig 2B), the lower donor strain B transfers slightly more to its kind than to K12, although the difference is not statistically significant. This fits more with a model of specificity in transfer than with a general correlation between donor and recipient ability (where B recipient ability would be expected to be lower).

Discrimination arising from specific interactions between genotypes that we just described cannot be studied from our model, as we assumed for simplicity that transfer is a product of individual donor ability $q_{\mathrm{ij}}$ and recipient ability $s_{\mathrm{ij}}$. Modeling specificity in transfer would require a set of interaction parameters that modulate transfer for each couple of donor and recipient strains, which goes beyond the scope of our study.
